# The Influence of Ectopic Migration of Granule Cells into the Hilus on Dentate Gyrus-CA3 Function

**DOI:** 10.1371/journal.pone.0068208

**Published:** 2013-06-28

**Authors:** Catherine E. Myers, Keria Bermudez-Hernandez, Helen E. Scharfman

**Affiliations:** 1 NeuroBehavioral Research Laboratory, Veterans Affairs Medical Center, New Jersey Healthcare System, East Orange, New Jersey, United States of America; 2 Neurology and Neurosciences, New Jersey Medical School, University of Medicine and Dentistry of New Jersey, Newark, New Jersey, United States of America; 3 Department of Psychology, Rutgers University, Newark, Newark, New Jersey, United States of America; 4 Sackler Program in Biomedical Sciences, New York University Langone Medical Center, New York, New York, United States of America; 5 Center for Dementia Research, the Nathan Kline Institute for Psychiatric Research, Orangeburg, New York, United States of America; 6 Child & Adolescent Psychiatry, Physiology & Neuroscience, and Psychiatry, New York University Langone Medical Center, New York, New York, United States of America; McGill University, Canada

## Abstract

Postnatal neurogenesis of granule cells (GCs) in the dentate gyrus (DG) produces GCs that normally migrate from the subgranular zone to the GC layer. However, GCs can mismigrate into the hilus, the opposite direction. Previous descriptions of these hilar ectopic GCs (hEGCs) suggest that they are rare unless there are severe seizures. However, it is not clear if severe seizures are required, and it also is unclear if severe seizures are responsible for the abnormalities of hEGCs, which include atypical dendrites and electrophysiological properties. Here we show that large numbers of hEGCs develop in a transgenic mouse without severe seizures. The mice have a deletion of *BAX*, which normally regulates apoptosis. Surprisingly, we show that hEGCs in the BAX^-/-^ mouse have similar abnormalities as hEGCs that arise after severe seizures. We next asked if there are selective effects of hEGCs, i.e., whether a robust population of hEGCs would have any effect on the DG if they were induced without severe seizures. Indeed, this appears to be true, because it has been reported that BAX^-/-^ mice have defects in a behavior that tests pattern separation, which depends on the DG. However, inferring functional effects of hEGCs is difficult in mice with a constitutive *BAX* deletion because there is decreased apoptosis in and outside the DG. Therefore, a computational model of the normal DG and hippocampal subfield CA3 was used. Adding a small population of hEGCs (5% of all GCs), with characteristics defined empirically, was sufficient to disrupt a simulation of pattern separation and completion. Modeling results also showed that effects of hEGCs were due primarily to “backprojections” of CA3 pyramidal cell axons to the hilus. The results suggest that hEGCs can develop for diverse reasons, do not depend on severe seizures, and a small population of hEGCs may impair DG-dependent function.

## Introduction

In the mammalian brain, GCs are born throughout life, a process called postnatal neurogenesis [1-9]. They are generated from precursors in the subgranular zone of the DG, and normally migrate a short distance to the adjacent GC layer (GCL), where they stop migrating, and develop characteristics that are remarkably similar to GCs born in early development. For example, the morphology, innervation by the perforant path, and the characteristics of the axons of adult-born GCs are similar to GCs born early in life [1-6]. Adult-born GCs also innervate the same cell types in the hilus and CA3 that are targeted by GCs born in development [1,3,4]. Once they mature, adult-born GCs have similar intrinsic properties and synaptic potentials to GCs born in early life. However, they have a critical period during their maturation – at approximately 4-6 weeks of age - when they exhibit increased excitability and plasticity compared to mature GCs [8-11].

In several animal models of temporal lobe epilepsy (TLE), a dramatic increase in DG neurogenesis has been reported as epilepsy develops, which is usually followed much later by a decline in DG neurogenesis [12-17]. In most of these animal models, the initial increase in adult neurogenesis occurs shortly after an experimental manipulation, which typically is injection of a convulsant to initiate several hours of severe continuous seizures (status epilepticus; SE). After 3-4 days, there is a substantial increase in proliferation in the DG subgranular zone, and many of these new cells become GCs that migrate to the GCL (GCL GCs). A large number of the GCs also migrate to the adjacent hilus, where they form an “ectopic” population (hilar ectopic GCs; hEGCs [12,17]). It has been suggested that SE causes hEGCs to form because it leads to excitotoxic cell death of hilar reelin-expressing neurons in the days after SE. The reduction in reelin, which is a “stop” signal for migrating neurons, causes newborn neurons in the subgranular zone to migrate to the hilus instead of the GCL [18].

HEGCs develop some characteristics of GCL GCs, such as a “mossy fiber” axon, which has a unique trajectory in stratum lucidum of area CA3, and unusually large (“giant”) boutons which occur at a specific periodicity along the parent mossy fiber [19-21]. In addition, intrinsic properties of hEGCs, recorded intracellularly in hippocampal slices after SE, are generally similar to GCs [17], although one study showed a depolarized resting potential in hEGCs compared to GCL GCs [22]. Other hEGC characteristics are distinct from GCL GCs, such as a bipolar dendritic tree (instead of an apical dendritic tree), although some hEGCs do have a dendritic arbor that is primarily apical [17,23,24]. Many hEGCs have spontaneous rhythmic bursts of action potentials, which are not observed in normal GCs [17,23,24]. Because of the abnormal dendrites and burst discharges of most hEGCs, it has been suggested that hEGCs could have adverse effects on the DG network, contributing to increased predisposition to spontaneous seizures after SE [14,25-32]. HEGCs may adversely influence DG-dependent cognitive functions also, which is important because hippocampal-dependent memory impairment is a significant comorbidity in patients with TLE [33,34]. Importantly, hEGCs have been reported in resected hippocampus removed from individuals with pharmacoresistant TLE [35], suggesting that what is found in the animal model is relevant to human epilepsy.

Another animal model of TLE that exhibits a large population of hEGCs uses experimental febrile seizures induced in early life (postnatal day 11 [36]), by raising body temperature to approximately 40^o^C for 30 min [36]. The experimental febrile seizures initiates changes in the brain which ultimately lead to seizures, simulating a syndrome in children who have severe (“complex”) febrile seizures and develop TLE later in life [37]. It has been shown that experimental febrile seizures impair normal GABAergic mechanisms that are responsible for migration of GCs during development, leading to mismigration of GCs into the hilus, i.e., hEGC formation [36]. On the basis of these findings, it has been suggested that hEGCs contribute to seizure susceptibility later in life, and explain the impairment in hippocampal-dependent behavior at that time [36].

These studies have raised an important question: are hEGCs only relevant to epilepsy? Might they develop under other conditions where migration is altered, or postnatal neurogenesis is abnormal? In these instances, would hEGCs lead to impairment of DG-dependent functions? Would hEGCs be sufficient to impair DG function, if it were possible to examine them selectively? These questions have been hard to answer because robust numbers of hEGCs have not been reported in the absence of epilepsy, and the selective effects of hEGCs are difficult to dissociate from effects of epilepsy.

Here we present the results of parallel empirical and computational modeling studies that were conducted to address these questions. First, we examined a BAX^-/-^ mouse to determine if hEGCs developed. *BAX* is one of the critical regulators of programmed cell death in development, so we predicted that *BAX* deletion would lead to the survival of DG progenitors. Since DG progenitors are present in high numbers in the hilus in early life, we predicted that if these progenitors survived, they might become hEGCs. We present results showing that the BAX^-/-^ mouse indeed develops a robust population of hEGCs.

We then asked what characteristics hEGCs would have in BAX^-/-^ mice and found, surprisingly, that they have morphological and physiological properties that are similar to those of hEGCs in epileptic animals. The data suggest that the unusual characteristics of hEGCs after SE or febrile seizures that were reported previously, and assumed to be caused by the SE or febrile seizures, actually can occur without them.

The next experiments addressed the ability of hEGCs to cause impairments in the DG network in BAX^-/-^ mice. Indeed, *BAX* ^*-/-*^ mice do have defects in contextual conditioning at 6 months of age [38]. However, *BAX* ^*-/-*^ mice have many abnormalities besides hEGCs [38-41]. These additional abnormalities make it hard to distinguish the effects of hEGCs selectively. Therefore, a computational model of the normal DG and CA3 regions, described previously [42,43], was used. A DG-dependent function was simulated that is often tested in rodents with a contextual conditioning task: pattern separation and completion. The ability of the model to simulate data from empirical tests of pattern separation and completion was confirmed [42,43]. Next, hEGCs were added to the simulated DG-CA3 network in numbers and with properties based on characteristics observed empirically – i.e., characteristics common to rats that have had SE and BAX^-/-^ mice. The modeling results suggest that hEGCs impair DG-dependent function. Taken together, the results underscore the importance of normal migration in the DG.

## Methods

### I: Empirical studies

Experiments were conducted in accordance with guidelines of the National Institutes of Health and New York State. They were approved by the IACUC of The Nathan Kline Institute. Reagents were purchased from Sigma-Aldrich (St. Louis, MO) unless otherwise stated.

#### A: Animals

BAX^-/-^ mice on a C57BL/6J background that have been previously characterized [41] were purchased from Jackson Laboratories (Bar Harbor, ME) at 1.5 months of age and allowed to acclimate for approximately 2 weeks under standard conditions (12 hr light: dark cycle, food and water *ad libitum*) before use. Animal care and use met the guidelines of the National Institutes of Health and New, York State Department of Health, and procedures were approved by The Nathan Kline Institute Animal Care and Use Committee.

#### B: Electrophysiology

Animals were deeply anesthetized by isoflurane (Aerrane; Henry Schein, Melville, NY) and then decapitated. The brain was rapidly removed and immersed in a slurry of sucrose-based artificial cerebrospinal fluid (ACSF; in mM: 252 sucrose, 3.5 KCl, 2.0 MgSO_4_, 2.0 CaCl_2_, 1.25 NaH_2_PO_4_, 26.0 NaHCO_3_, 10.0 d-glucose). After approximately 1 min, a hemisphere was trimmed with a razor blade so the dorsal surface was flat and that surface was glued with cyanoacrylate to the top of a tray that was immersed in ice-cold ACSF. Horizontal sections (400 µm) were cut with a Vibroslice (World Precision Instruments, Sarasota, FL), placed in a beaker containing oxygenated (95% O_2_/5% CO_2_) room temperature ACSF for 5-10 min, and then transferred by a wide-bore glass pipette to a nylon net of a recording chamber [17,44] where slices were perfused from below; all but the uppermost surfaces were covered. Temperature where the slices were located (30-31^o^C) was maintained by a feedback temperature controller (PTCO3, Scientific Systems Design, Mississauga, Ontario, CA). Warm, humidified (95% O_2_, 5% CO_2_) air was vented over the slice surfaces. Inflow rate (1 ml/min) was maintained by a peristaltic pump (Minipuls 2, Gilson, Middleton, WI). After 30 min, inflow was changed from sucrose-based ACSF to NaCl-based ACSF (126 mM NaCl). Recordings began 30 min later.

Recording electrodes were pulled from borosilicate glass (0.75 mm inner diameter, 1.0 mm outer diameter; World Precision Instruments, Sarasota, FL) using a horizontal pipette puller (P87; Flaming-Brown, Sutter Instruments, Novato, CA) and were 60-70 megaohms when filled with pre-filtered (0.2 µm; Thermo, Fisher Scientific, Morristown, NJ) 4% Neurobiotin (Vector Laboratories, Burlingame, CA) in 1.0 M potassium acetate. Intracellular recordings were made with an amplifier with a bridge circuit (Axoclamp 2B, Molecular Devices, Sunnyvale, CA) and the bridge was balanced whenever current was passed. The stimulating electrode was a monopolar Teflon-coated stainless steel wire (75 µm-diameter, including Teflon; A-M Systems, Carlsborg, WA). Stimuli were controlled by a stimulus isolator (AMPI, Jerusalem, Israel) triggered by pClamp (Molecular Devices). Data were acquired using a Digidata 1440 (Molecular Devices) in pClamp and a computerized oscilloscope (Model Pro10, Nicolet, Madison, WI). Neurobiotin was injected after acquiring electrophysiological data, using methods described elsewhere [17,44].

Analysis used pClamp and OriginPro (Originlabs, Northampton, MA). Intrinsic properties and characterization of firing were conducted as previously described [17,44]. A spontaneous burst was defined as a depolarization that evoked 1-3 action potentials and occurred without electrical stimulation or current injection. Burst frequency was determined from at least 30 sec of continuous record; this duration was chosen because it greatly exceeded the interburst interval.

#### C: Anatomical procedures

After recording, slices were transferred with a wide-bored pipette to ACSF and then 4% paraformaldehyde in 0.1 M phosphate buffer (PB) overnight at 4^o^C. Slices were then laid onto a Petri dish and drops of warm 4% agar were placed on them. Each slice, with the agar around it, was then placed in 2% paraformaldehyde in 0.1 M PB overnight at 4^o^C. Agar was removed from the area around the slice, and then the slice was glued to the surface of a 4% agar block and resectioned on a vibratome (75 µm). Sections were washed in 0.1 M Tris buffer, incubated in 0.25% Triton-X 100 in Tris buffer, incubated in ABC (Standard ABC kit; Vector) for 2 hrs, and reacted in diaminobenzidine as described elsewhere [44].

Procedures that were used for experiments besides those where electrophysiology was conducted using hippocampal slices were as follows. Animals were deeply anesthetized with isoflurane by inhalation, followed by an overdose of urethane (2.5 g/kg i.p.). After opening the abdominal cavity, a 26g butterfly needle was inserted into the heart, directed towards the aorta. The heart was first infused with 0.9% NaCl with a peristaltic pump (Minipuls 2, Gilson, Middleton, WI) and then immediately perfused with 4% paraformaldehyde (pH 7.4). The brain was removed and post-fixed in 4% paraformaldehyde for at least 24 hrs. Sections (50 µm) were cut in the horizontal plane with a vibratome (Model TPI1000; The Vibratome Company, St. Louis, MO) in 0.1 M Tris buffer.

Free-floating sections were processed as previously described [45]. In brief, sections were first placed in 0.2% Triton X-100, then blocked for 30 min in normal goat serum (5%; Vector) for rabbit antibodies (Prox1, neuropeptide Y; NPY) or normal horse serum (5%; Vector) for mouse antibodies (NeuN, an antibody against a neuronal nuclear antigen). After washing in Tris buffer, sections were incubated overnight in a rabbit polyclonal antibody to Prox1 (1:10,000; EMD Millipore, Billerica, MA), NPY (1:5,000; Immunostar, Hudson, WI) or NeuN (1:5,000; EMD Millipore). Then sections were incubated for 45 min in secondary antibody, which was biotinylated goat anti-rabbit IgG for rabbit antibodies (1:400; Vector) or biotinylated horse anti-mouse IgG for mouse antibodies (1:400; Vector). Next, sections were incubated for 2 hrs in ABC (Standard kit; Vector), and reacted in diaminobenzidine (for details see 44) in 5 mM NiCl_2_.

For Prox-1/NeuN double-labeling, sections were stained with Prox1 first, and then blocked with normal horse serum (5%; Vector) followed by incubation overnight with a mouse monoclonal antibody to NeuN. Sections were incubated with secondary antibody and reacted with NovaRed (Vector) according to the manufacturer’s instructions.

Sections were coverslipped in Permount and photographed with a brightfield microscope (Model BX61, Olympus of America, Center Valley, PA) and digital camera (RET-2000; Q Imaging, Surrey, British Columbia) using ImagePro software (Media Cybernetics, Bethesda, MD). Cells were counted as double-labeled if the nucleus and cytoplasm were brought into focus simultaneously at 80X magnification, i.e., they were located in the same focal plane. Cells were designated as hilar if the edge of the cell body closest to the GCL was at least 10 µm from the nearest edge of a soma of a GC in the GCL. The GCL was defined by GCs that were located adjacent to each other (i.e., there was no detectable intercellular space between them). The hilus was defined as zone 4 of Amaral [46,47].

### II: Computational modeling studies

**Figure 1:**
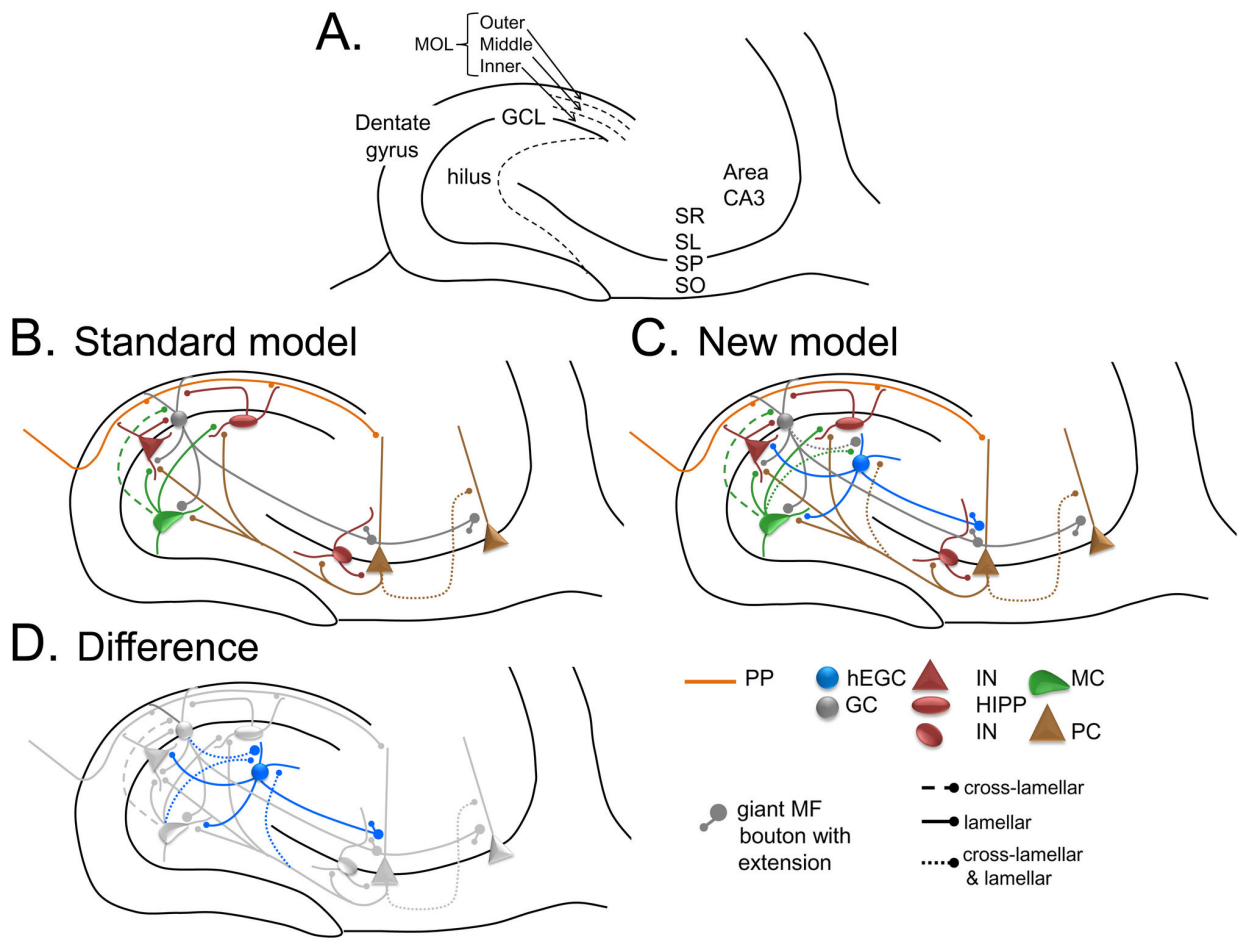
Circuitry of the DG-CA3 network. **A**. A schematic of the DG and area CA3 is shown. The DG is composed of three layers: the molecular layer (MOL; divided into thirds corresponding to the outer, middle and inner molecular layers), the GC layer (GCL), and the hilus. Area CA3 includes several layers, such as stratum radiatum (SR), stratum lucidum (SL), stratum pyramidale (SP; also called the pyramidal cell layer), and stratum oriens (SO). **B**. The circuitry of the normal dentate gyrus, used for the Standard model. There are several simplifications of the known circuitry; rationales for simplificationsare provided elsewhere [42,43]. The major cell type in the DG module of the model is the GCL GC (grey). GCL GCs have dendrites in the molecular layer and an axon in the hilus and CA3. Dendrites receive afferent input from the perforant path in the outer and middle molecular layers (orange) and input from hilar mossy cells in the inner molecular layer (green). Pathways that are lamellar in orientation are indicated by solid lines; pathways that are cross-lamellar are shown as dashed lines; pathways that are both lamellar and cross-lamellar are designated by dotted lines. GC axons are called ‘mossy fibers’ and collateralize in the hilus where they make small boutons on interneurons (INs) and large boutons on mossy cells. The main axon projects to SL of CA3 where it innervates CA3 INs and pyramidal cells. Filamentous extensions of large boutons in SL innervate CA3 GABAergic IN (red). The large boutons innervate pyramidal cells. Other cell types in the DG include hilar mossy cells and GABAergic INs. Mossy cells have hilar cell bodies and dendrites in the model. They receive input from the GCL GCs and pyramidal cells. Their axon is simplified in the model to include only the primary projection: local innervation of INs and distal innervation of GCL GCs. INs are simplified to represent the two primary types: INs that innervate the soma or axon hillock of GCs (perisomatic-targeting) and INs that innervate the dendritic region. The cell in the figure that represents the perisomatic-targeting cell type is a triangular red cell, corresponding to the most common cell type of this class, the pyramidal basket cell. In the model, it receives input primarily from GCL GCs and inhibits GCL GCs in the same lamella. The cell representing a dendritic-targeting IN is the oval red hilar cell corresponding to the most common cell type of this class, the HIPP cell (a *hi*lar cell body and a projection to the terminal zone of the *p*erforant *p*ath). The HIPP cell receives input from the perforant path and inhibits GCL GCs in the same lamella. Together these two types of INs represent not only perisomatic and dendritic-targeting INs, but also feedforward and feedback inhibition. In addition to phasic inhibition, tonic inhibition is also incorporated in the model by making the threshold for GCL GC activation relatively high. In the model, pyramidal cells give rise to a divergent axon, targeting local INs, other pyramidal cells (by recurrent collaterals innervating pyramidal cell dendrites in SR), and there is a backprojection to the DG that targets mossy cells and DG INs. The CA3 IN represents both feedforward and feedback inhibition, and provides both perisomatic and dendritic inhibition of pyramidal cells. **C**. The New model. The New model represents a modification of the Standard model as follows: 1) a small number of hEGCs (blue) are added, with dendritic arbors restricted to the hilus, 2) hEGCs receive input from GCL GC mossy fiber ‘giant’ boutons, mossy cell input, and pyramidal cell backprojection input, 3) hEGC dendrites are oriented both within and across lamellae; because of the dendritic orientation, the CA3 input is similar to the recurrent collateral system, both within and across lamellae, and 4) hEGCs make a mossy fiber axon that is similar to GCL GCs. **D**. The differences between the Standard and New models are shown with hEGCs in blue and other cells light gray. The differences are: 1) hEGC cell bodies and dendrites in the hilus, 2) hEGC axons to CA3 and mossy cells, 3) GC mossy fiber input to hEGCs, and 4) input from CA3 backprojections to hEGCs.

The DG-CA3 model is based on previous studies of DG and CA3 circuitry which is schematically diagrammed in Figure 1. The “Standard” DG-CA3 computational model is similar to that previously described in Myers & Scharfman [42,43] and is based on a DG network that interacts with a CA3 network, with cell types, connectivity patterns, and firing properties based on known features of the DG and CA3 circuitry; simplifying assumptions and other factors governing cell numbers and properties in the model are described in previous publications [42,43]. Aside from the use of a sigmoidal activation function, and some additional anatomical characteristics of the CA3 backprojection described below, the “Standard” model is the same as previously described [42].

This “Standard” model was then altered to form a “New” model that included adult-born (immature) hEGCs, in numbers and with properties that were common to previous studies of hEGCs in SE-induced epilepsy [17,48-53] and hEGCs of the BAX^-/-^ mice described in the Results. For comparison, an “Intermediate” model was also constructed that contained the same number of adult-born (immature) GCs as the New model, but all immature GCs were located in the GCL rather than the hilus. A brief description of the “Standard” DG and CA3 networks, and of the “Intermediate” and “New” models is provided below, with full simulation details in the Text S1.

#### A: “Standard Model”: DG network

The DG network includes a GCL with 1000 GCs, divided into 10 simulated lamellae. The DG network receives as input a 100-element vector representing axonal projections from the lateral perforant path, which carries sensory information as well as input from the medial perforant path, which carries spatial information. Each vector of perforant path inputs is a "pattern" that can be stored and/or recognized by the model. Each GC fires (generates action potentials) in response to that pattern if the weighted sum of perforant path inputs, inhibition from local interneurons, and excitatory inputs from mossy cells and other sources, passed through a sigmoidal function, exceeds a firing threshold for that GC. Because GCs have a relatively high resting potential and high threshold for action potential generation *in vitro* [44,54-57] and firing rate is low *in vivo* compared to other hippocampal neurons [58,59], the GC threshold in the model is set fairly high (0.75). Aside from the use of a sigmoidal activation function, the DG network is as previously described [42].

Interneurons are divided into two primary groups in the DG model; first, those that target the perisomatic region of GCL GCs (e.g., the pyramidal basket cell), represented by a triangular red DG cell in Figure 1. One of the inputs to this interneuron type in the model is GCL GCs within the same lamella. The axon of the interneuron is local (in the same lamella as the GCs that activated it) and “feeds back” to inhibit GCL GCs in the same lamella. Second, the DG network also includes GABAergic neurons that target GC dendrites; in the model, this category is represented by the so-called HIPP cell (***hi***lar cell body, axon projection in the terminal zone of the ***p***erforant ***p***ath [60]). In the model, the axons of HIPP cells project to GC dendrites; the DG model simplifies several aspects of both interneuron types, as discussed previously [42,43].

The DG model also includes glutamatergic hilar cells called mossy cells; consistent with prior empirical data, the mossy cells receive input from GCL GCs within the same lamella and send excitatory projections to GCL GCs outside that lamella. In addition, mossy cells innervate local interneurons with processes in the hilus, represented by lamellar input to the dendrites of perisomatic-targeting interneurons and HIPP cells (Figure 1C). Therefore, mossy cells can inhibit GCL GCs indirectly via interneurons in the same lamella, and excite GCL GCs outside the lamella by direct excitatory input. In the model, the distribution of extralamellar (synonymous with cross-lamellar) mossy cell input to GCs is similar in density across all lamella outside the mossy cell body, because of evidence that mossy cells in the ventral hippocampus in the mouse have axon projections that appear to be similar in density throughout the septotemporal axis [61]; dorsal mossy cells may differ, however, as reviewed elsewhere [46].

Output from the GCs forms the mossy fiber pathway, which has a dense projection to hilar neurons (mossy cells, HIPP cells, hilar dendrites of perisomatic-targeting interneurons; Figure 1B) and area CA3. Mossy fibers make giant boutons which primarily innervate the proximal dendrites of CA3 pyramidal cells and mossy cells, and also have extensions that primarily innervate interneurons in area CA3, only one of which is shown in Figure 1B.

#### B: CA3 network

The CA3 network includes 300 pyramidal cells, divided among the 10 lamellae like the DG. Within a given lamella, pyramidal cells receive dendritic input from the perforant path, recurrent collaterals from other pyramidal cells (not confined to the same lamella), and mossy fibers (Figure 1B).

The mossy fiber input to pyramidal cells is very strong, based on empirical data (electron microscopy) showing that the giant boutons that innervate pyramidal cells are densely packed with glutamatergic vesicles; furthermore, these inputs have a very large quantal size and have been called detonators [62]. In the network, these giant boutons act as "teaching inputs" that depolarize pyramidal cells at the time of perforant path input, leading to greater synaptic plasticity [63-65].

Pyramidal cells fire (generate action potentials) if the weighted sum of all excitatory and inhibitory inputs, passed through a sigmoidal activation function, exceeds a threshold (0.5). This threshold is lower than GCs because of intrinsic properties that lead to discharge in bursts even at threshold [66]. In contrast, there is only one action potential in GCs at threshold [17].

Output of pyramidal cells is sent to other pyramidal cells (via the recurrent collaterals), and to local interneurons which feed back to inhibit the majority of pyramidal cells in the same lamella. Therefore, both feedforward and feedback inhibition exist in the model, although the multitude of cell types that mediate inhibition of pyramidal cells are simplified to one cell type in the model (Figure 1B).

Following Myers & Scharfman [43], the model also includes a projection from pyramidal cells to the DG hilus (‘backprojections’; [67]). In Myers & Scharfman [43], the backprojection was modeled so that it inhibited GCs in the same lamella, and did so relatively simply (i.e., direct inhibitory connections from pyramidal cells to GCs in the same lamella). In the current iteration of the DG-CA3 model, we have made the backprojection more consistent with known anatomy, i.e., the pyramidal cell axons innervate mossy cells and interneurons, leading to inhibition of GCs that is both disynaptic (pyramidal cell → DG IN → GC) and polysynaptic (e.g., pyramidal cell → mossy cell → DG IN → GC), consistent with empirical data [68-72].

Notably, it has been shown that direct pyramidal cell → GC projections exist, particularly in the temporal hippocampus [72]. However, the robust nature of the backprojection-mediated inhibition of GCs in recordings from slices [68] and observations that many pyramidal cells do not have axons that cross the GCL [69,73], suggest that inhibition predominates within a given hippocampal lamella. Outside the lamella of origin of a given pyramidal cell, however, effects of the backprojection on GCs are likely to be primarily excitatory, for two reasons. First, pyramidal cells innervate mossy cells in the same lamella, and mossy cells project outside the lamella and the primary effect is to excite GCs. Second, the interneurons in the DG that pyramidal cells innervate have axons that primarily innervate GCs within the same lamella. There are reports of cross-lamellar inhibition of GCs [74-44], but the primary effect of inhibitory neurons, in general, is local.

#### C: Incorporating adult-born neurons in the computational model

**Figure 2:**
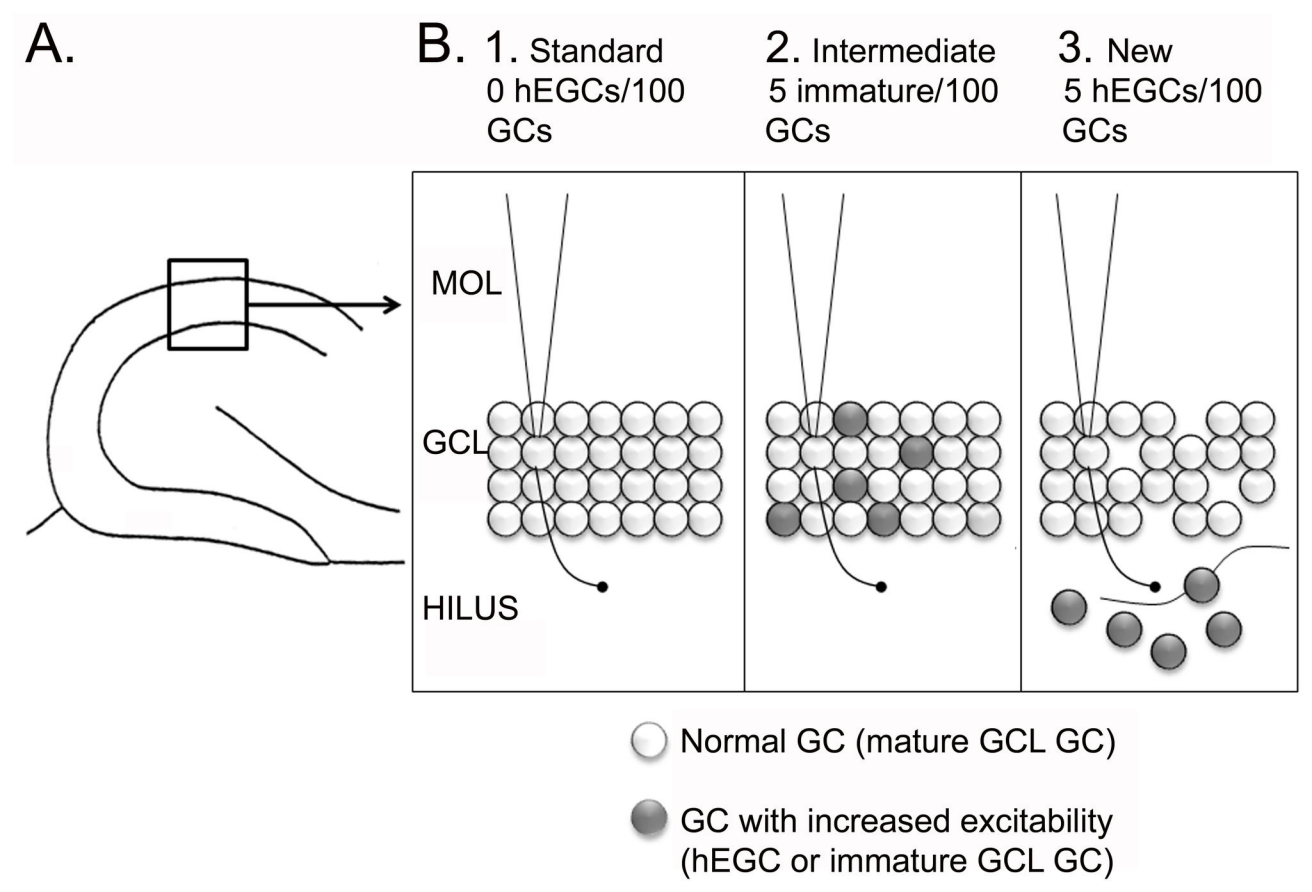
Three computational models of the DG-CA3 network. **A**. A box outlines the area of the DG shown in B. **B**. The three computational models are shown. 1. Standard model. There are 1000 GCs and all are mature with relatively high thresholds/low excitability. MOL = molecular layer. GCL = granule cell layer. 2. Intermediate model. For every 100 GCs, 5 are adult-born neurons that are immature. They have increased excitability relative to the other 95 GCs, which are mature. 3. New model. For every 100 GCs, 5 are located in the hilus. They have increased excitability relative to the other 95 that are mature.

In Figure 2, the three versions of the DG-CA3 model that were used for simulations are shown. **Standard** refers to the DG-CA3 model of Figure 1B, where adult neurogenesis is not considered. The ***Intermediate*** model incorporates adult neurogenesis by making 5% of the GCs in the GCL (GCL GCs) adult-born (“immature”); the other 95% are mature. The estimate of 5% is based on previous quantitative studies of postnatal neurogenesis in rodents, showing that approximately 4-10% of the GCL GCs in the adult mouse are immature GCs [1-6] at any one point in adulthood. Previous electrophysiological studies of immature GCs suggest that they are more excitable than GCL GCs because of their membrane properties, increased propensity for LTP, and depolarizing response to GABA [8-11]. Therefore, the immature GCs in the Intermediate model have a firing threshold of 0.7, lower than the value (0.75) for mature GCL GCs. Inputs and outputs of immature GCs in the model are otherwise the same as mature GCs, based on the available empirical data [1-6].

The third model, called “***New***,” incorporates hEGCs into the DG network. It is similar to the Intermediate model except that the 5% of immature GCs are located in the hilus, not the GCL (Figure 1C). The characteristics of the hEGCs in the New model were based on hEGCs in rats that have SE [17,49-53] and BAX^-/-^ mice (described below).

#### D: Data collection and analysis

To examine behavior of the network, the model was provided with sets of randomly-constructed 200-bit patterns, with each pattern constrained to have a fixed percentage *d* of its elements active (=1) and the remainder inactive (=0). These patterns were presented as perforant path inputs to the DG and CA3 networks, where they contacted cell types as shown in Figure 1.

As in Myers & Scharfman [42,43], the model operates in two phases. In the first phase, perforant path input is triggered and pyramidal cells are activated. Then the activity is allowed to influence other pyramidal cells by recurrent collaterals. This process allows the CA3 network to reactivate “patterns” of CA3 activity that have previously been stored in response to the same (or similar) perforant path inputs – a process called pattern completion. Information is also allowed to flow from CA3 to the DG (via the “backprojection”) and DG cells are allowed to become active and produce output (action potentials). At this point, the percentage of GCs and pyramidal cells firing (producing action potentials) can be calculated.

Following the “pattern completion” phase, a “pattern storage” phase occurs, in which CA3 recurrent collaterals are silenced but the mossy fiber pathway from the GCs to CA3 is not. The mossy fibers produce large depolarizations of CA3 pyramidal cells, which facilitates the storage of the perforant path input pattern in CA3. As in Myers & Scharfman [42,43], synaptic strengths (weights) of perforant path inputs to pyramidal cells and recurrent collaterals to pyramidal cells are modified by Hebbian-like learning, as a function of conjoint pre- and post-synaptic activity. This process allows pattern storage in CA3.

As in Myers & Scharfman [42,43], to test pattern separation, the average Hamming distance (HD) metric is used. For a given set of *p* patterns of perforant path input, the differences or ‘distance’ between two patterns is defined as the number of elements that differ between the two patterns. The average HD can be computed for a set of input patterns, independent of the model components, and can also be computed for GC (and pyramidal cell) responses to those inputs. If the average HD computed for GC (or pyramidal cell) responses is greater after training than it was initially (when input patterns were first presented), then pattern separation is said to have occurred in the DG (or CA3).

To test pattern completion, a set of *n* input patterns is provided to the model, and each input pattern has d=10% of the elements active (corresponding to action potentials in 10% of the perforant path inputs). The model undergoes 10 passes through this training set, with pattern completion and pattern storage phases for each pattern occurring during each pass. Next a series of test patterns are constructed, which represent “distorted” versions of the trained patterns; these are constructed by setting a fixed percentage (*e*=0-90%) of the active elements in each pattern to 0. Ten such “distorted” versions of each trained pattern are created, where *p* is the trained pattern and *p*’ is the distorted pattern. Each distorted pattern *p*’ is then presented to the model, which undergoes a pattern completion phase, allowing pyramidal ceβlls to become active. The pattern of pyramidal cell activity in response to *p*’ can be compared to the pattern of pyramidal cell activity in response to the original, trained pattern *p*; if the overlap is greater for *p* and *p*’ than for any other trained pattern, then the network has successfully performed pattern completion.

Model data are reported as the average of 10 simulation runs, except for the exceptional case where the Standard model, Intermediate model, New model, and New without backprojections were compared For this case, the results are presented for one simulation run for each model type (Standard, Intermediate, New and New without backprojections), averaged across 10 patterns. Error bars represent ± standard error of the mean (s.e.m.). Variation in model results occurs because, at the start of each simulation run, the DG and CA3 networks are initialized with new cell-to-cell connectivity matrices and synaptic weights. For example, in one simulation run, a particular pyramidal cell might receive recurrent collaterals from a particular subset of pyramidal cells inside the same lamella, and others outside the lamella; on a later simulation run, the same cell might receive recurrent collaterals from a different subset of the pyramidal cell population. In addition, there also is variance because pattern sets differ for each simulation run; pattern sets are constructed randomly, so they differ, but they are constructed according to a fixed rule (e.g., percentage of active elements). Because of this variance, we subjected the simulation results to statistical analysis - similar to the analysis used to examine empirical data - to determine whether observed group differences were statistically significant, or could simply be due to the way the model behaved for a particular set of input patterns that were not representative of the mean sampling error based on variability in initial conditions or trained patterns. Analogous to the manner that statistics would be used for empirical data of this kind, data were analyzed using univariate or mixed analysis of variance (ANOVA), followed by Bonferroni-corrected post-hoc *t*-tests for pairwise comparisons. Analysis was conducted using SPSS v. 19 (IBM Corporation, Armonk, NY).

## Results

### I: BAX^-/-^ *mice*

#### A: hEGCs in *BAX* ^*-/-*^ mice

**Figure 3:**
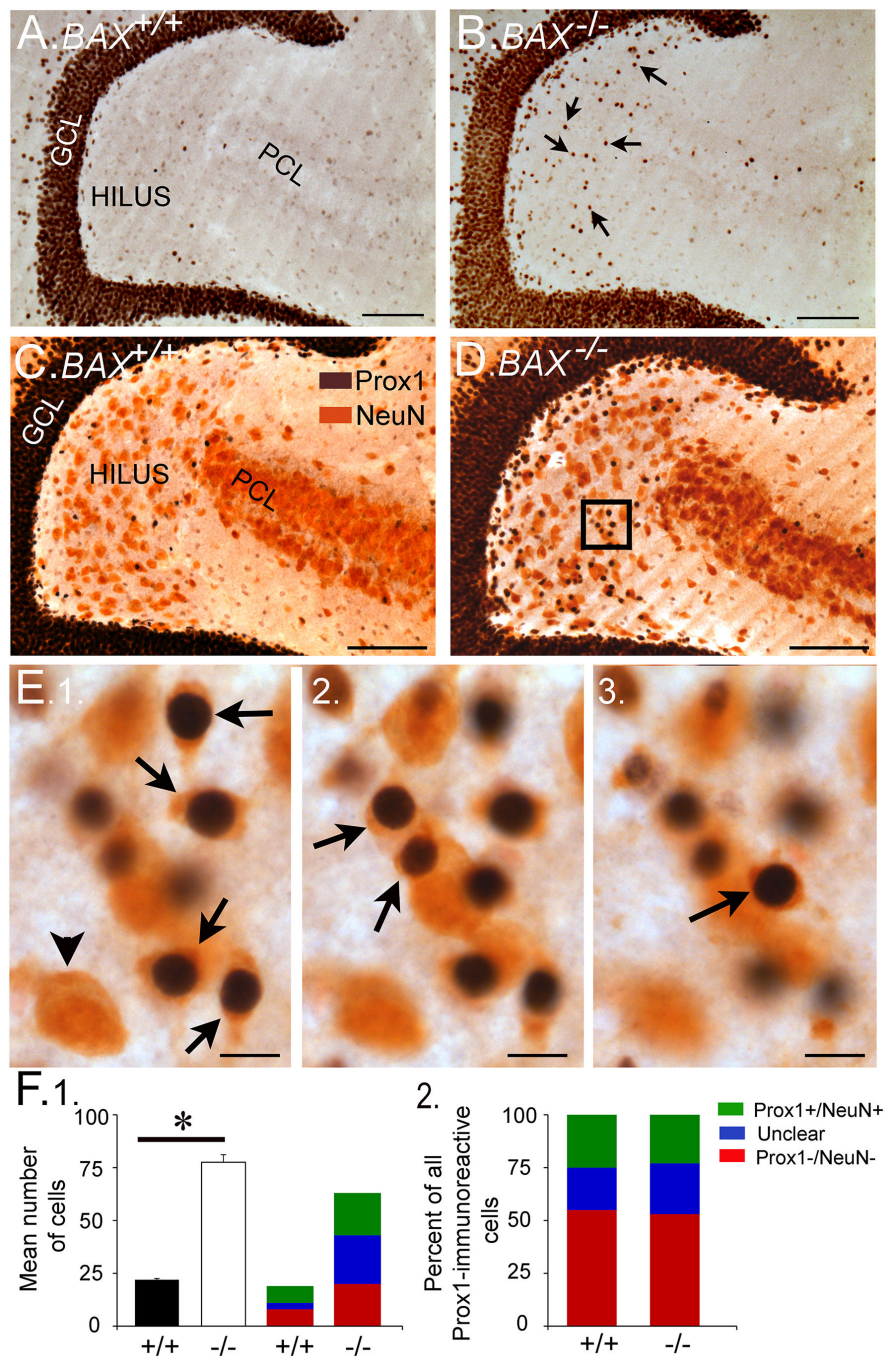
Prox1-ir cells in BAX^+/+^ and BAX^-/-^ mice. **A-B**. Representative horizontal sections illustrate Prox1-ir nuclei in the hilus of a BAX^+/+^ (A) and BAX^-/-^ mouse (B) at 2 months of age. Arrows point to Prox1-immunoreactive nuclei in the hilus. GCL = granule cell layer. PCL = CA3 pyramidal cell layer. Calibration in A-D = 100 µm. **C-D**. Prox1 and NeuN labeling in a BAX^+/+^ and BAX^-/-^ mouse (D). The area surrounded by the box is enlarged in E. **E**. 1-3. Double-labeled cells (Prox1+/NeuN+) are indicated by the arrows. Prox1 labels the nucleus and NeuN labels the cytoplasm, which come into focus in the same focal plane when the cell is double-labeled. Successive focal planes are shown in 1-3. This area corresponds to the boxed area in D. The arrowhead denotes a NeuN+ cell that was not labeled by Prox1. Calibration = 15 µm. **F**. Quantification of the Prox1-labeled and NeuN-labeled cells. 1. The mean number of double-labeled hilar cells in BAX^-/-^ mice (white bar) was significantly greater (*p*<0.05; asterisk) than double-labeled hilar cells in BAX^+/+^ mice (black bar). The mean numbers of double-labeled (Prox1+/NeuN+; green) and Prox1+/NeuN-cells (red) are also shown. There also was a subset of Prox1+ cells that could not be classified definitively (as NeuN+ or NeuN-) because they were located in a cluster of overlapping Prox1-ir cells, or the cytoplasm showing NeuN-ir was small. 2. There were similar proportions of Prox1+ cells that were NeuN+, regardless of the genotype.

To our knowledge, the only sizeable population of hEGCs that has been reported to date is in epileptic rodents after SE or febrile seizures [17,36,48,79]. Here, we report that BAX^-/-^ mice develop a population of hEGCs that is robust. The mice were examined at 2 months of age (n=8 BAX^-/-^; n=7 BAX^+/+^ controls). As shown in Figure 3A-B, Prox1-immunostained nuclei were abundant in the hilus of BAX^-/-^ mice compared to controls. For quantification, the numbers of Prox1-ir cells in a subset of mice were counted (n=3 BAX^+/+^; n=3 BAX^-/-^ mice). The mean number of Prox1-ir cells was over 3x greater in BAX^-/-^ mice (77.9 ± 3.6 Prox1-ir cells/section) compared to BAX^+/+^ mice (21.9 ± 0.5 Prox1-ir cells; *t*-test, *p*<0.01).

Most Prox1-ir cells co-expressed NeuN and therefore were neurons. Out of the total number of Prox1-ir cells, 52.7 ± 8.2% were double-labeled with NeuN in BAX^-/-^ mice and 55.0 ± 3.2% in BAX^+/+^ mice, which was not a statistically different subset (*t*-test, *p*>0.05; Figure 3C–F). These values are estimates; the actual values of double-labeled cells could be higher, because of two types of possible double-labeling that were hard to assess and therefore, to be conservative, were designated “unclear” (Figure 3C–F). One type of this possible double-labeling occurred when there were clusters of Prox-1 nuclei in the subgranular zone, which occurred primarily in BAX^-/-^ mice. In these clusters, overlapping nuclei were surrounded by NeuN-labelled cytoplasm but individual cells were difficult to discriminate. This primarily occurred in BAX^-/-^ mice. The second type of equivocal double-labeling occurred when little cytoplasm surrounded a Prox-1-ir nucleus, which is common in GCL GCs, where the large nucleus often is only surrounded by a thin rim of cytoplasm [82,83]. Regardless, a substantial number of Prox1-ir cells were neurons, based on clear Prox1-ir profiles that co-expressed NeuN (Figure 3C–F).

#### B: Characterization of hEGCs in *BAX* ^*-/-*^ mice

**Figure 4:**
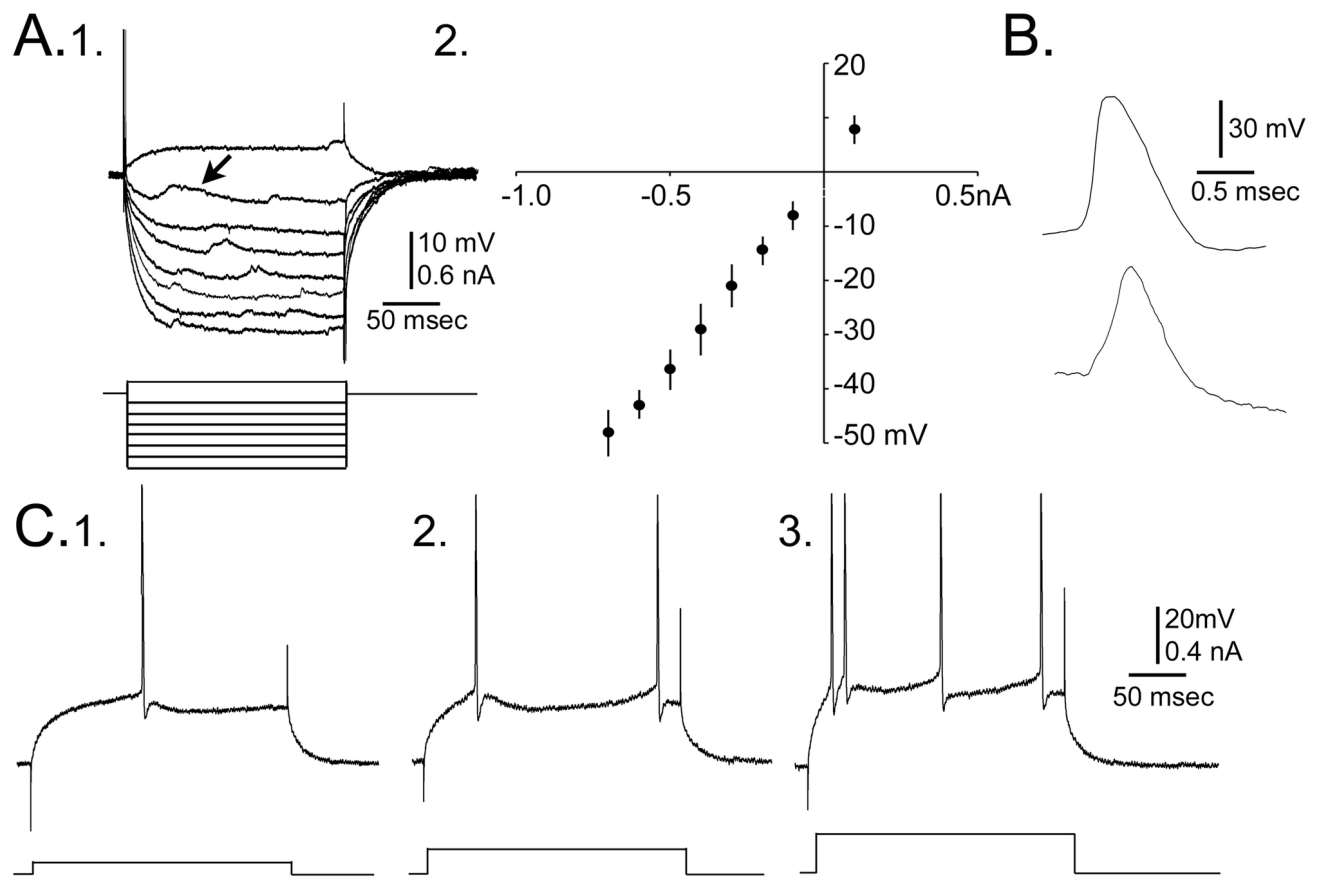
Intrinsic properties of hEGCs in BAX^-/-^ **mice**. **A**. 1. Superimposed responses of a hEGC to depolarizing and hyperpolarizing current steps at resting potential (-70 mV). Arrow marks spontaneous synaptic potentials. 2. The amplitude of voltage responses at steady state to current steps is plotted for all hEGCs (mean ± s.e.m.), illustrating that hEGCs had linear I–V curves, similar to GCL GCs [17,54,55]. **B**. Top: a representative hEGC action potential at threshold showing a steep rate of rise relative to rate of decay, similar to GCL GCs [17,44]. Bottom: an example of a typical action potential of an interneuron, illustrating a slower rate of rise of the action potential relative to the rate of decay, typical of interneurons [17,44]. **C**. A representative example of hEGC responses to 3 depolarizing current steps at -65 mV holding potential. Current amplitude increases from left (1) to center (2) and is highest in (3). Firing behavior is similar to a GCL GC in that there is no bursting (clusters) of spikes and there is spike frequency adaptation [17,44].

**Figure 5:**
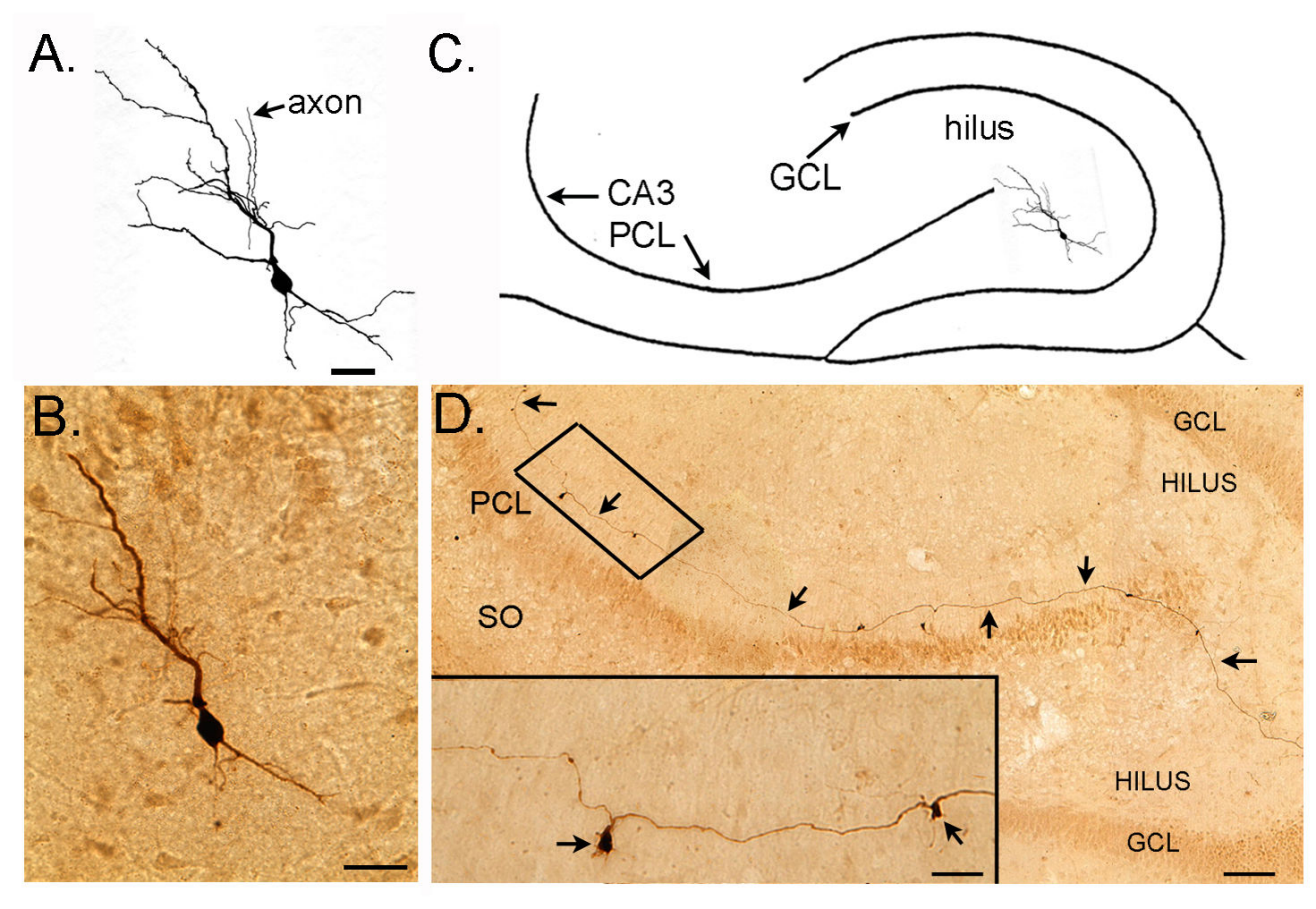
Intracellularly-labeled hEGC from a BAX^-/-^ mouse. **A**. A drawing of a Neurobiotin-filled hEGC. The cell body is a similar size and shape to a GCL GC. Arrows point to axon segments. Calibration = 15 µm. **B**. A montage of the Neurobiotin-filled hEGC in A. Calibration = 20 µm. **C**. The cell in A is drawn in the location and with the orientation it had in the hippocampal slice where it was recorded. **D**. A montage illustrates the mossy fiber axon of the hEGC. Arrows point to the axon, which entered CA3 at the border of the hilus and CA3, and then coursed parallel to the pyramidal cell layer (PCL). The area of the axon that is inside the box is shown at higher power in the inset. Calibration = 100 µm. Inset: mossy fiber giant boutons (arrows). Calibration = 10 µm.

**Figure 6:**
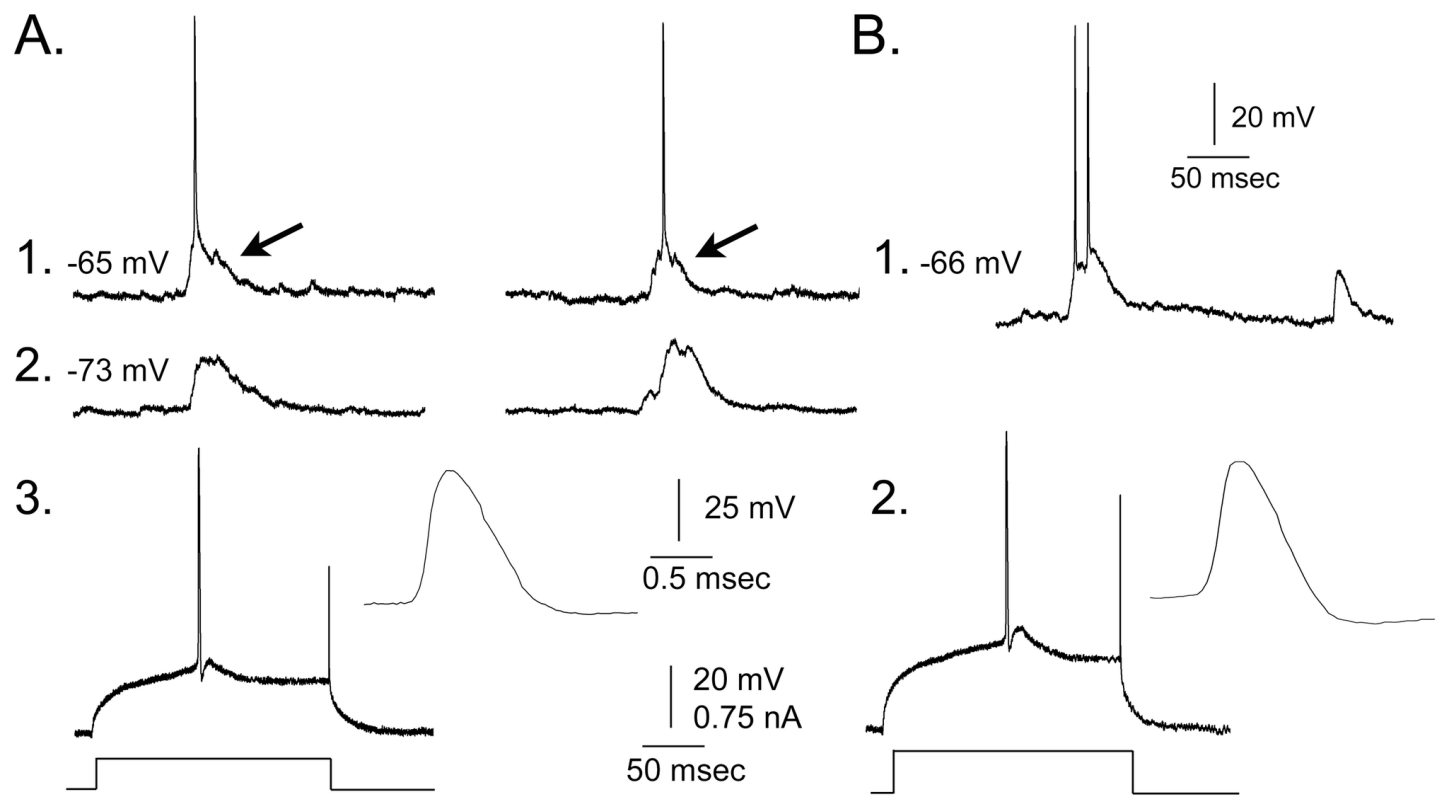
Spontaneous bursts of action potentials of hEGCs of BAX^-/-^ mice. **A**. Spontaneous depolarizations and action potentials (arrows) in a hEGC. 1. Holding potential, -65 mV. 2. Holding potential, -73 mV. 3. An action potential evoked by intracellular current injection at threshold for the hEGC in A. Inset: the action potential shows a typical steep rate of rise relative to decay, and duration similar to a GC [17,44]. **B**. 1. Spontaneous depolarizations in a different hEGC. Holding potential, -66 mV. 2. Action potential evoked at threshold.

Hippocampal slices were used to characterize hilar cells. Numerous cells in the hilus of BAX^-/-^ mice had electrophysiological characteristics of normal GCs (10 of 34 cells in 8 slices from 4 BAX^-/-^ mice, 2 months old; Table 1). The electrophysiological characteristics were 1) a relatively hyperpolarized resting potential (-70 to -80 mV), 2) relatively short time constant (<15 msec), 3) lack of rectification in the response to a 200 msec current pulse (Figure 4), 4) regular spiking firing pattern (AP duration, >1 msec; Figure 4), 5) spike frequency adaptation (increasing interspike interval for a current pulse over threshold; Figure 4), and 6) a triphasic afterhyperpolarization following a single action potential [17,44,46,55] (Table 1 Figure 4).

**Table 1:** Intrinsic properties of hEGCs in BAX^-/-^ mice.

		Resting potential (mV)	Input resistance (megahoms)	Time constant (msec)	Action potential amplitude (mV)	Action potential duration (msec)
hEGCs	Mean	72.1	91.3	14.6	101.8	1.5
	SEM	1.0	2.5	1.0	2.9	0.1
	N	10	10	10	10	10

Intrinsic properties of cells in the hilus of slices from BAX^-/-^ mice had electrophysiological properties similar to GCL GCs. The measurements and other details of methods are provided in the text and elsewhere [17.44].

Intracellular injection of Neurobiotin into 3 hilar neurons with electrophysiological characteristics of normal GCs showed that their morphology was similar to GCs in past studies [21,27,49,80-83]. For example, these neurons had cell bodies that were similar in size (~10 µm) and shape (round or oval) to GCs, spiny dendrites like GCs, and their axons were similar to mossy fibers in that the main branch traveled to area CA3 and coursed through stratum lucidum in a relatively straight path, making periodic large boutons with filamentous extensions (Figure 5). Basal and apical dendrites were both present, similar to hEGCs in animal models of epilepsy [27,49,80]. The mossy fiber collateralized in the hilus, where some large boutons were also located, and there were many smaller terminals on axon collaterals in the hilus, which is typical of mossy fibers (Figures 5, 19-21).

All other hilar neurons had electrophysiological characteristics of mossy cells or interneurons [17,44,46,55] In BAX^-/-^ mice, these were a subset of all hilar cells (BAX^-/-^ : 24 of 34 cells, 8 slices, 4 mice) whereas in BAX^+/+^ mice all of the hilar cells were either mossy cells or interneurons, based on electrophysiological critieria (32 of 32 cells, 7 slices, 4 mice, all 2 months old).

#### C: hEGCs in BAX^-/-^ mice exhibit spontaneous discharges

One of the notable characteristics of hEGCs in previous studies of epileptic rats was spontaneous intermittent bursts of action potentials, which occurred in the majority of hEGCs [17; see also 22]. This characteristic was remarkable because normal GCL GCs do not exhibit spontaneous action potentials [17,55,58,59]. The burst discharges of hEGCs in epileptic rats were attributed to the epileptic tissue, where pyramidal cells developed rhythmic bursts; it was suggested that pyramidal cells drive hEGCs by their backprojections [17,25,26,49,52,79].

Several hEGCs of 2 month-old BAX^-/-^ mice exhibited intermittent spontaneous discharges also (Figure 6). The frequency of spontaneous discharges was 0.10-0.25 Hz (range; mean ± sem, 0.16 ± 0.04 Hz; n=4 hEGCs), similar to previous studies of hEGCs in epileptic rats (range: 0.05-0.28 Hz; mean ± sem: 0.17 ± 0.02 Hz, n=12 hEGCs; *t*-test, *p*=0.732 [17]). The burst discharges of hEGCs in BAX^-/-^ mice were usually short (range: 36-50 msec; mean ± sem: 44.00 ± 7.12 msec) relative to those described before in epileptic tissue (40-410 msec; 136.67 ± 98.47 msec; *t*-test, *p*=0.077 [17]). Only 1-2 action potentials were triggered in bursts from hEGCs of BAX^-/-^ mice (Figure 6), whereas bursts could have more than 2 action potentials in hEGCs of epileptic rats [17].

**Figure 7:**
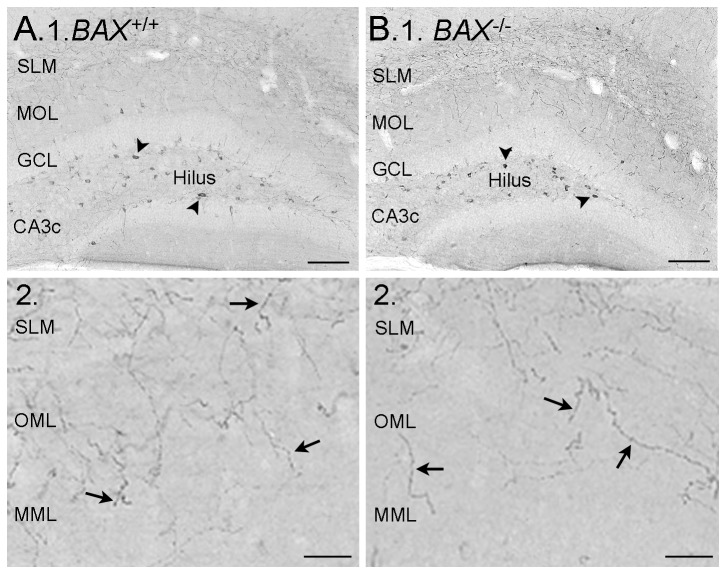
HEGCs from BAX^-/-^ mice have normal neuropeptide Y (NPY) staining. **A**. 1. NPY-ir in a dorsal coronal section from a BAX^+/+^ mouse shows numerous hilar neurons express NPY (arrowheads) but not GCL GCs. MOL = molecular layer. SLM = stratum lacunosum-moleculare of area CA1. Calibration = 100 µm. 2. A higher magnification illustrates the fibers in the outer molecular layer (OML) and middle molecular layer (MML; arrows), corresponding to axons of HIPP cells, the most common type of NPY-expressing cell that projects to the molecular layer. Calibration = 25 µm. **B**. 1. A section from a similar part of the hippocampus as A1, in a BAX^-/-^ mouse. Same calibration as A1. 2. A higher magnification of the molecular layer in B1 shows NPY-ir fibers, similar to the BAX^+/+^ mouse. Same calibration as A2.

These data suggested that BAX^-/-^ mice contained hEGCs and the hEGCs could exhibit spontaneous burst discharges. Because of the similarity of these observations to the epileptic rodent, we considered the possibility that BAX^-/-^ mice had seizures. In previous studies of BAX^-/-^ mice, seizures were not reported [38-41], but they could have been missed because seizures may have occurred when mice were not monitored. We addressed the possibility that BAX^-/-^ mice had seizures using an antibody to neuropeptide Y (NPY), which labels mossy fibers in rats that have spontaneous recurrent seizures [84-88]. Two month-old BAX^-/-^ mice (n=4) did not exhibit NPY-ir in mossy fibers (Figure 7). NPY labeling was similar to wild type mice (n=3), with numerous cell bodies labeled in the hilus, reflecting the large number of NPY-expressing cells there, corresponding to HIPP cells [89]. NPY-ir fibers were primarily in the outer and middle molecular layer in both BAX^-/-^ and BAX^+/+^ mice (Figure 7), similar to previous descriptions [90,91].

The results from BAX^-/-^ mice suggested that hEGCs can develop without SE or febrile seizures. Moreover, characteristics of hEGCs in BAX^-/-^ mice were similar to hEGCs in epileptic rats. Common characteristics of hEGCs were 1) similar intrinsic properties to normal GCs, 2) similar anatomical characteristics to normal GCs (soma size, soma shape, spiny dendrites, mossy fiber axon) and 3) more excitability than normal GCs, reflected by a predisposition to discharge in spontaneous rhythmic bursts.

### II: Computational modeling

#### A. Incorporation of hEGCs into the computational model

To form the “New” model (Figures 1C-D, 2B2) we added hEGCs to the computational model with characteristics of increased excitability relative to GCL GCs, specifically by reducing the firing threshold from 0.75 to 0.7 (i.e., same as immature GCL GCs in the Intermediate model). The increase in excitability was intended to reflect the fact that many hEGCs develop spontaneous burst discharges.

Inputs to hEGCs in the New model (Figure 1C-D) were based on previous studies of hEGCs in the epileptic rat as follows: hEGCs receive excitatory mossy fiber input from GCL GCs, input from mossy cells, and input from CA3 backprojections [17,49-52]. In the New model, hEGC dendrites are confined to the hilus (Figure 1C-D), so they are not innervated by perforant path or interneurons. This is a simplification, since HIPP and other GABAergic neurons have axons in the hilus, but seems reasonable in light of the fact that perisomatic –targeting and HIPP cells primarily innervate the GCL and molecular layer [60,91,92]. Also, there is dense excitatory input to hEGC dendrites [23,27,49,50] and greater ratio of excitatory to inhibitory input in recordings from hEGCs [24]. hEGCs with dendrites in the molecular layer are located close to the GCL based on previous studies [23,53], and therefore could be considered a part of the GCL, so they are excluded from the model for simplicity.

Recently it was shown that the dendrites of hEGCs in the epileptic rat extend along the longitudinal as well as transverse axis of the hippocampus [49,53]. Therefore, in the New model, hEGCs receive input from pyramidal cells both within the same lamella and from other lamellae (Figure 1C-D). We assume that the mossy fiber axon of hEGCs is similar to normal GCL GCs because the empirical data suggest that [17] (Figure 5) – i.e. projecting to mossy cells within the same lamella as well as to pyramidal cells and interneurons within the same lamella (Figure 1), and making strong (giant bouton) synapses onto pyramidal cells [19-21,93].

Note that in animal models of epilepsy, hEGCs make a major contribution to mossy fiber sprouting [17,49,94], which refers to the growth of new collaterals from the parent mossy fiber to the inner molecular layer [95,96]. BAX^-/-^ mice did not exhibit sprouting of mossy fibers, based on NPY-ir as a mossy fiber marker (Figure 7). For these reasons, mossy fiber sprouting is not included in the New model.

#### B: HEGCs influence the DG response to the perforant path input

To test pattern separation, the model was trained on a small set (n=10) of randomly-constructed patterns, with each pattern constrained to have d=10% of its elements active (i.e., 10% of the elements in each pattern were set to 1 and the rest to 0).

**Figure 8:**
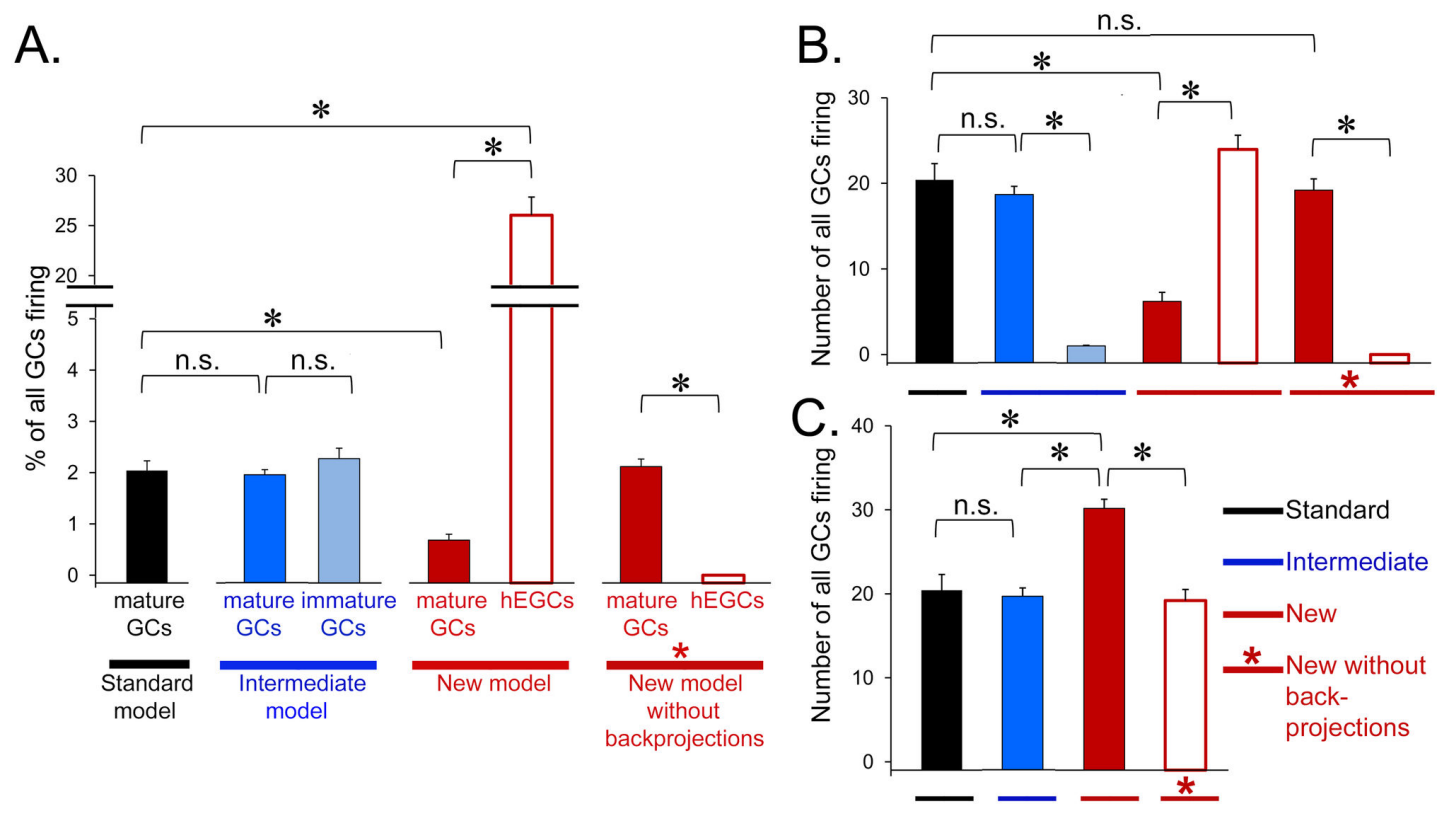
Effects of hEGCs on action potential generation of GCs. Results are shown from a single representative simulation run with the Standard (black), Intermediate (blue) and New (red) models. **A**. To determine effects of the perforant path on GC action potential generation (firing), 10 patterns, each with 10% active elements, were presented as input (from the perforant path). The GCs that were active (discharged action potentials), averaged across patterns, are expressed as a **percentage** of each GC subtype. For the Standard model (black), a small percentage (about 2%) of mature GCs became active in response to the input patterns, consistent with the relatively high threshold of mature GCs. In the Intermediate model (blue), a similar percentage of mature (dark blue) and immature GCs (light blue) became active (all, *p*>0.50; n.s. = not significant; asterisks denote *p*<0.05). A smaller percentage of mature GCs in the New model (dark red) were active compared to the Standard or Intermediate models (*p*<0.05). hEGCs in the New model (white outlined in red) were very active, significantly more than the mature or immature GCs in the Standard or Intermediate models; hEGCs were also more active than mature GCs in the New model (*p*<0.0001). When CA3 backprojections were removed from the New model (red with asterisk), hEGCs (white outlined in red) were effectively silenced, and firing of mature GCs (dark red) was restored to the activity of mature GCs in the Standard model (*p*>0.50). **B**. GC firing, expressed as the **absolute** number of each GC subtype. Most results were similar to part A. The only difference was the relative activity of mature and immature GCs in the Intermediate model. In part A, there were similar percentages of mature and immature GCs that were active, but in absolute numbers (B), there were significantly more active mature GCs than active immature GCs in the Intermdediate model. The reason for the similarity in percentages – but not absolute numbers - is that the number of active GCs is always small relative to all GCs. **C**. Total GC firing, expressed as the **absolute** number of all GCs (the sum of mature, immature, and hEGCs). Total GC firing did not differ significantly among Standard, Intermediate, and the New model without backprojections (all *p*>0.100), but was significantly higher in the New model with backprojections (*p*<0.0001).

In the Standard model, where all GCs are located in the GCL (Figures 1B, 2B1), the number of GCs that produce mossy fiber output (“GC activity”, below) in response to such input patterns is low, with ~2% of all GCL GCs firing on average to any given input pattern (Figure 8A, Standard Model).

In the Intermediate model (Figures 1C, 2B3), where 5% of GCs are immature GCs, both mature and immature GCs fire in response to perforant path input (Figure 8A-B, Intermediate Model). However, the percentage of mature GCs that are active does not significantly change from the percentage observed in the Standard model (*t*-test, *p*>0.050), and there are so many more mature relative to immature GCs in the Intermediate model that the total number of GCs that fire is not significantly different in the Standard and Intermediate models (Figure 8C; *p*>0.050).

In the New model (Figure 2B2), where hEGCs are 5% of the GC population, the hEGCs show robust firing in response to perforant path input - much stronger than mature or immature GCL GCs in the Standard and Intermediate models (Figure 8A, New model). The greater response of hEGCs relative to other GCs is interesting because hEGCs do not have a stronger direct perforant path input in the model. However, they do have much stronger excitatory input from GCL GCs, unlike mature and immature GCs. They also have a relative lack of inhibitory input (Figure 1C-D).

Interestingly, there was a decrease in activity of the mature GCL GCs in the New model compared to the Standard model (Figure 8A-B; *t*-test, *p*<0.0001), implying that adding a small population of hEGCs to the DG network produces an inhibitory effect on GCL GCs (discussed further below). However, so many of the hEGCs are activated that the total number of GCs firing is greater in the New model than in the Standard or Intermediate models (Figure 8C; both *p*<0.001) despite the inhibition of GCL GCs in the New model.

An advantage of computational modeling is that it is possible to explore which of the features of hEGCs in the model are most responsible for observed effects selectively. One would think that the primary determinant of hEGC firing would be activity in GCL GCs because of the powerful mossy fiber boutons of GCL GCs that innervate hEGCs. However, when CA3 backprojections are removed from the model (Figure 8A; New model without backprojections), hEGCs are silenced -- and the activity of mature GCL GCs is restored to the level observed in the Standard model (Figure 8A,C; *t*-tests; *p*<0.0001). These data suggest that CA3 backprojections have a very important role when hEGCs are present.

#### C: HEGCs impair pattern separation in the DG

**Figure 9:**
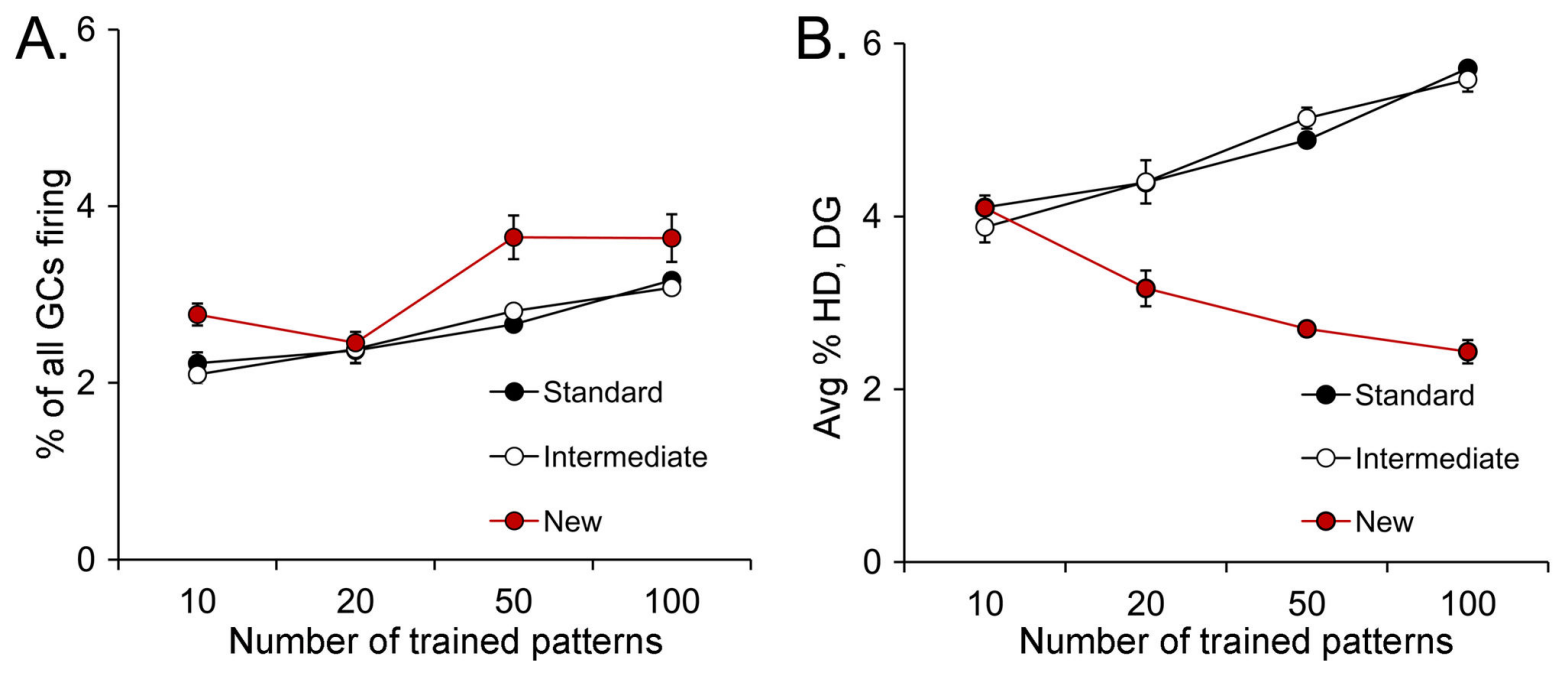
Effects of hEGCs on pattern separation in the DG. **A**. Effects of increasing input patterns on GC firing in the Standard, Intermediate and New models. Increasing input patterns had similar effects in the Standard and Intermediate models, which did not exhibit a large increase in GC firing as pattern number increased. There was a modest effect in the New model, but it was statistically different when compared to the other models (repeated measures ANOVA, *p*<0.05). **B**. Effects of input patterns on average % Hamming distance (Avg % HD) is used as a reflection of pattern separation in the DG. In response to increasing input patterns, the Avg % HD increased in the Standard and Intermediate models, but decreased in the New model with hEGCs.

The next question we asked was how GC firing was affected as the number of trained patterns increased from 10 (Figure 8) to 20, 50, or 100 (Figure 9A). Remarkably, the activity of GCs (all GCs, averaged) in the three models (Standard, Intermediate and New) was fairly stable when the number of trained patterns increased from 10 to 20, 50, or 100 patterns (Figure 9A). There was a slight increase in overall GC activity in the New model, presumably reflecting the high activity of hEGCs (as shown in Figure 8A–C). Although the effects in Figure 9A are small, there was a significant increase in GC firing for an increased number of stored patterns (*F*(3,81)=31.53, *p*<0.001) as well as a difference between models (*F*(2,27)=15.97, *p*<0.001). Specifically, the New model differed significantly from the Standard and Intermediate models (both *p*<0.001) but the Standard and Intermediate models did not differ from each other (*p*>0.500).

When trained on a set of 10 patterns, the degree of pattern separation in the DG (reflected by Avg % HD) was similar for the Standard model and the Intermediate model, and increased slightly as the number of stored patterns increases from n=10 to n=100 (Figure 9B). However, the New model showed **reduced** pattern separation as n increased above 10 (Figure 9B). ANOVA confirmed these impressions, revealing both a significant increase in Avg % HD as number of patterns increased (*F*(3,81)=7.13, *p*<0.001) as well as a difference between models (*F*(2,27)=144.56, *p*<0.001); there was also a pattern x model interaction (*F*(6,81)=22.58, *p*<0.001. Post-hoc comparisons confirmed that the New model differed significantly from the Standard and Intermediate models (both *p*<0.001), which did not differ from each other (*p*>0.500). Thus, a relatively small number of hEGCs (5% of the total GC population) is sufficient to degrade pattern separation in the New model, especially when the challenge (number of stored patterns) increases. Although this effect is dramatic in Figure 9B, it occurs without a large change in overall GC firing. One explanation is that the persistent firing of a small number of GCs (the hEGCs) degraded pattern separation. This idea is based on the following assumptions: under normal conditions a very small number of GCs fire so a small number of additional hEGCs could be influential. Furthermore, for pattern separation to be robust, any GCs that do respond should not respond to all patterns. Therefore, persistent firing of a subpopulation of GCs (hEGCs) would degrade pattern separation. For these reasons, a small population of GCs (hEGCs) could make a difference to the network.

#### D: Effect of hEGCs on activity and pattern separation in CA3

**Figure 10:**
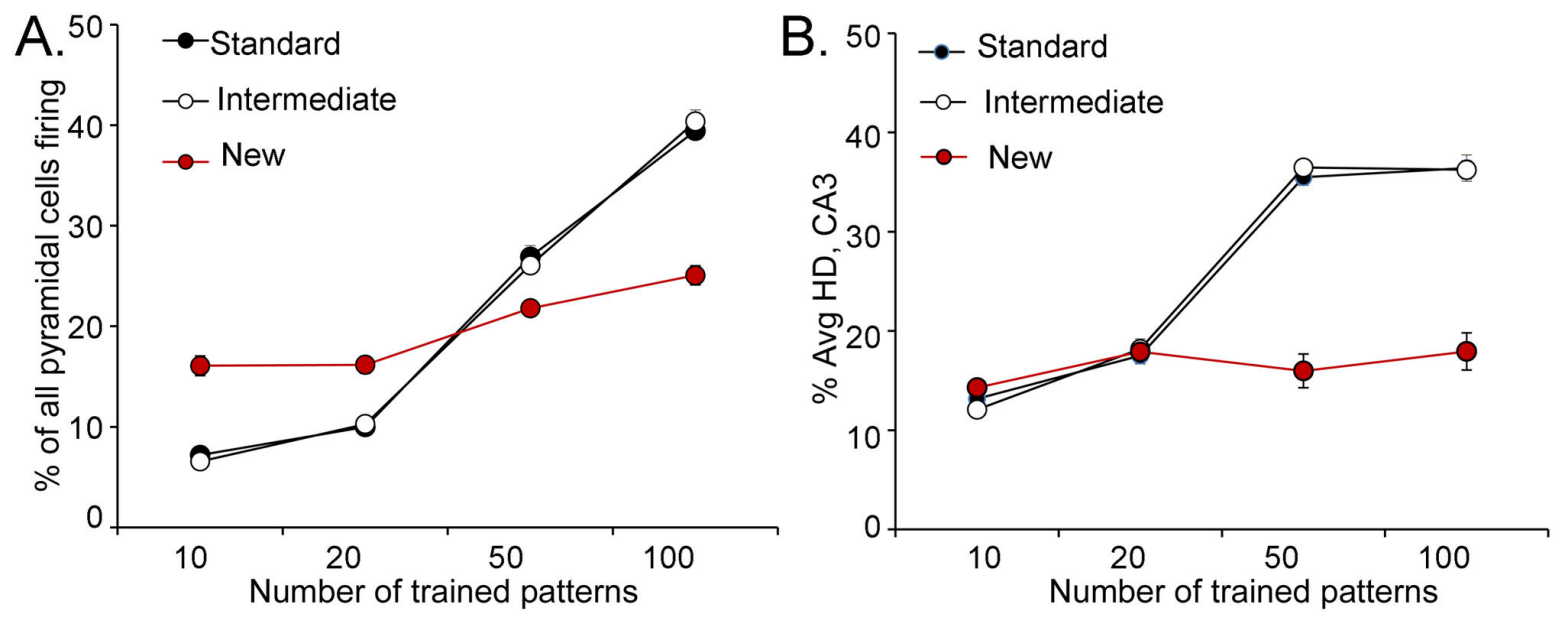
Effects of hEGCs on pattern separation in CA3. **A**. The effects of perforant path input patterns on CA3 pyramidal cell firing was tested in the three models. Increasing the number of input patterns increased the firing of pyramidal cells in the Standard and Intermediate models, but there was a normalization of firing in the New model with hEGCs. **B**. Experiments analogous to those in Figure 9B were conducted but the CA3 module was assessed instead of the DG module. In the Standard and Intermediate models, there was an increase in pattern separation of CA3 as the number of input patterns increased. However, as the number of input patterns increased, pattern separation by the New model was severely impaired.

The presence of hEGCs in the New model had a large influence on pyramidal pyramidal cell firing (Figure 10A). Specifically, the Standard and Intermediate models showed an increase in pyramidal cell firing with an increase in the number of trained patterns, but the New model did not. Thus, when the number of trained patterns was low, the New model showed greater pyramidal cell activity than the Standard and Intermediate models; when the number of trained patterns was high (50+), pyramidal cell firing was **lower** in the New model than in the other two models. ANOVA confirmed these impressions, revealing a significant effect of number of stored patterns (*F*(3,81)=984.11, *p*<0.001) and a pattern x model interaction (*F*(6,81)=96.07, *p*<0.001) but no main effect of model (*F*(2,27)=1.25, *p*=0.302).

Figure 10B shows that the effects of hEGCs on pattern separation in CA3 were similar to the effects of hEGCs on pattern separation in the DG. Thus, pattern separation in the CA3 network decreased when hEGCs were present, particularly as the number of stored patterns increased. ANOVA confirmed these impressions, revealing a significant effects of number of stored patterns (*F*(3,81)=189.14, *p*<0.001) and model (*F*(2,27)=98.52, *p*<0.001) as well as a pattern x model interaction (*F*(6,81)=39.98, *p*<0.001). Post-hoc comparisons confirmed that the New model with hEGCs differed significantly from the Standard and Intermediate models (both *p*<0.001) which did not differ from each other (*p*>0.500). In summary, pattern separation, measured in either DG or CA3, was significantly worse in the New model than in the Standard or Intermediate models.

#### E: hEGCs influence pattern completion in CA3

**Figure 11:**
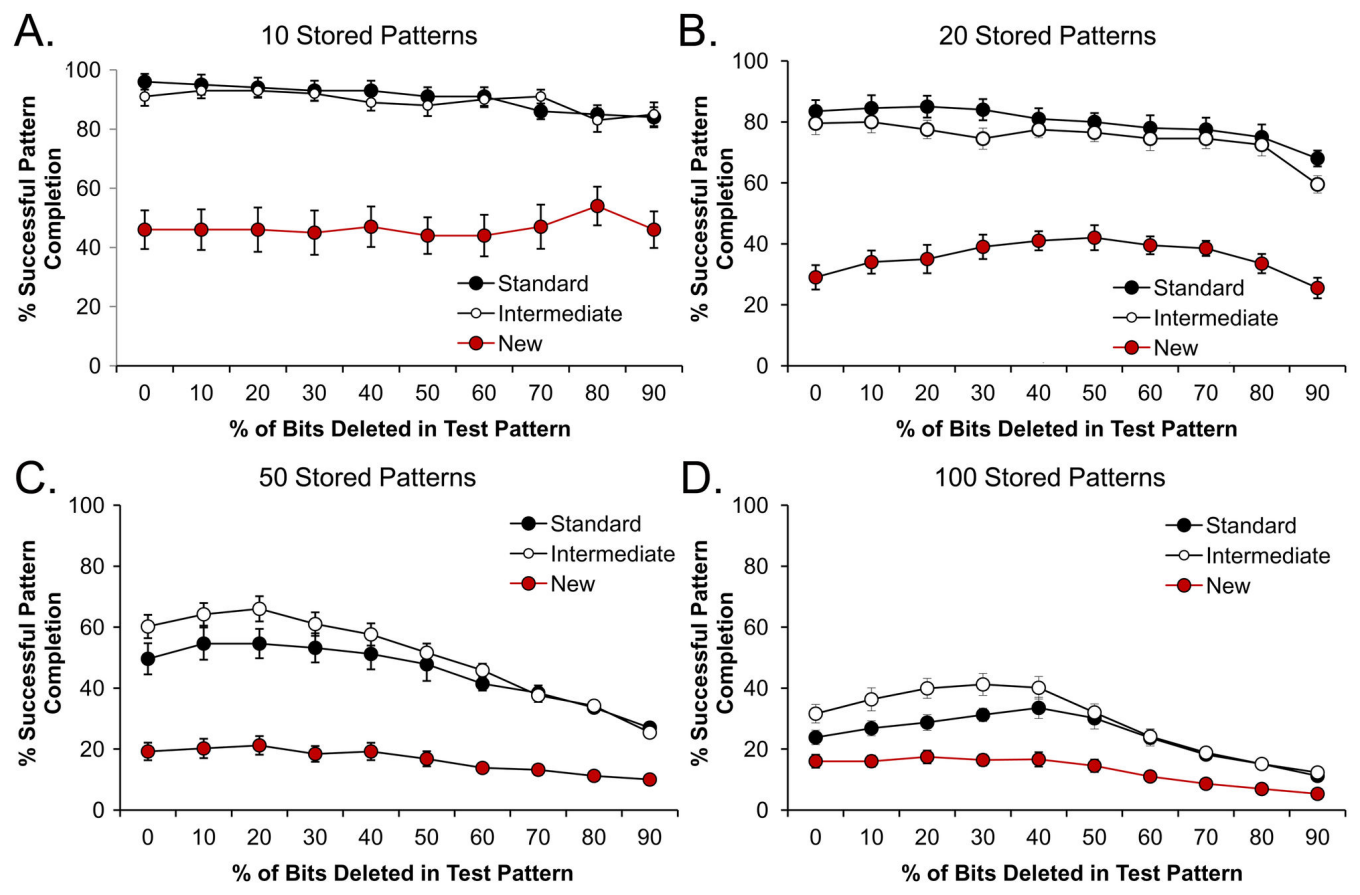
Effects of hEGCs on pattern completion in CA3. Pattern completion was assessed after training the model with specific input patterns, and then testing the model with input patterns that had varying degrees of deletion in the trained pattern. Pattern completion was defined as the percent of the tested trials where CA3 activity resembled the trained pattern. The degree of deletion is expressed on the X axis as the % of bits (e) deleted in the trained pattern. Results shown are averaged over 10 test trials, generated at each level of deletion (*e*=0%...100%) for each trained pattern. Results represent the average performance of the model over 10 identical iterations (simulation runs). **A**. Following training on a small set of 10 patterns, the Standard model showed good performance when each trained pattern was presented with *e*=0% deletion (i.e., test patterns identical to trained patterns). Performance degraded slowly as *e* increased. In contrast, the New model was severely impaired, even when tested on patterns with *e*=0% deletion (i.e., test pattern = trained pattern). **B-D**. As the number of trained patterns increased, the Standard and Intermediate models continued to perform well at small values of e, with a gradual decrease in performance as *e* increased. However, the New model was severely impaired. The Intermediate model was distinct from the New model, suggesting that simply increasing excitability in 5% of GCs is less influential than location in the hilus, and the associated effects of that location.

As described above, pattern completion was assessed by training the model on a set of 10 patterns and then administering distorted test patterns, i.e., with *e*=0-90% of the active elements deleted (Figure 11). Pattern completion was successful if the CA3 output was similar to the correct stored pattern relative to the other stored patterns. Figure 11A shows that, for a range of e, the Standard model was highly successful at pattern completion, almost always approximating the correct stored pattern even as *e* approached 90% (i.e., 90% of the trained pattern was omitted in the distorted version). Pattern completion performance was also good when the Intermediate model was used. However, pattern completion was markedly degraded in the New model with hEGCs. The degradation in performance was striking: for example, even when *e*=0% (the test pattern was identical to a trained pattern), the correct stored pattern was not always retrieved (Figure 11A).

In Figure 11A, the number of stored patterns was small (10) and performance was relatively stable. In Figure 11B–D, the number of stored patterns increased from 20, 50 and finally 100, and pattern completion in the Standard and Intermediate models became progressively worse, but the New model was much more severely affected. ANOVAs for pattern completion behavior using 10, 20, 50, and 100 patterns revealed significant effects of the model (all *F*>15.00, all *p*<0.001) and of the degree of deletion (e) in the test pattern (all *F*>14.00, all *p*<0.001), except for the 10-pattern condition where performance was relatively stable across all values of *e* (*F*(9,243)=2.28, *p*=0.018). There were also significant *e* x model interactions (all *F*>2, all *p*<0.002). In all cases, the New model performed significantly worse than the Standard and Intermediate models (all *p*<0.001) which did not differ (*p*>0.200). In summary, pattern completion was much worse in the New model than the Standard model and Intermediate models.

## Discussion

There are two primary findings of this study. First, hEGCs were identified in a transgenic mouse with a deletion in *BAX*, which is the first study to our knowledge that has described a robust population of hEGCs without using SE or febrile seizures to induce their development. The results suggest that there are multiple mechanisms to produce hEGCs. The implication is that hEGCs are not only relevant to epilepsy, but possibly other disorders where neuronal migration may be disrupted.

Interestingly, hEGCs in *BAX* ^-/-^ mice had characteristics similar to hEGCs described previously in epileptic rats that experienced SE. Therefore, the results suggest that hEGCs have similar anatomical and physiological properties, independent of the reason that they formed. Presumably a major stimulus to morphological and electrophysiological development of hEGCs is their location in the hilus, but many factors are likely to contribute.

The second primary finding of the study was that incorporation of hEGCs into a computational model of the DG-CA3 regions influenced the model in many ways, some of which were surprisingly large given the size of the hEGC population was small. The effects were mainly detrimental, suggesting that hEGCs, should they develop, would be likely to impair function.

The modeling results also suggest that backprojections from pyramidal cells play an important role in the net effects of hEGCs. This finding is consistent with empirical studies of rodents after SE, where spontaneous burst discharges of hEGCs in epileptic rats were caused by the backprojections [17,67,79]. The circuitry that involves the backprojections may be diverse: 1) pyramidal cell → hEGC (by direct input from backprojections), 2) pyramidal cell → pyramidal cell (by recurrent collateral excitation) → hEGC (by backprojections); or 3) pyramidal cell → mossy cell (by backprojections [1]) → hEGCs, (a pathway which has been demonstrated [51]). The modeling results thus support previous ideas that the backprojection is an important pathway to hippocampal-dependent functions in the normal rodent [67,72,97] and in epilepsy [67].

### I: HEGCs in BAX^-/-^ mice without SE or febrile seizures

There were many hEGCs in BAX^-/-^ mice as well as rodents after SE or febrile seizures. However, there were some differences. In the present studies of Prox1 labeling in BAX^-/-^ mice, many Prox1-ir nuclei did not double label with neuronal markers. Therefore, some Prox1 cells in *BAX* ^*-/-*^ mice are likely to be progenitors that have not yet committed to a neuronal fate, or non-neuronal cells.

The observation that hEGCs develop after a period of severe seizures (SE, febrile seizures) or *BAX* deletion suggests that there are multiple mechanisms that result in hEGCs. After SE, it had been proposed that hEGCs develop because SE causes vulnerable hilar somatostatinergic neurons to die [18]. Without a source of hilar reelin, GC progenitors migrate into the hilus from the subgranular zone [18]. Another mechanism to produce hEGCs was described in a study of febrile seizures: it was suggested that GABA_A_ receptors were upregulated in progenitors by febrile seizures and increased GABA-mediated depolarizations, which led to a reversal of the normal migratory path to the GCL [36]. Another mechanism that causes hEGC formation is infusion of recombinant BDNF into hippocampus, although the numbers of hEGCs were very low [98]. Nevertheless it is relevant because BDNF increases survival of adult-born GCs [99]. Therefore, both BDNF and *BAX* deletion may have a similar effect-increasing hEGC formation by increasing survival. However, there are seizures after BDNF infusion, and the seizures may have influenced migration as much as BDNF itself [98,100].

Regarding the mechanism of hEGC formation in BAX^-/-^ mice, it seems likely that a reduction in programmed cell death early in life contributed, because at this time of life numerous progenitors are located in the hilus. Without *BAX*, more of these progenitors in the hilus would be likely to survive, and those that stay in the hilus could become hEGCs. However, we cannot rule out the idea that some progenitors that exist in the hilus of adult BAX^-/-^ mice might also survive [13], or that progenitors in the adult subgranular zone survive and mismigrate, and contribute to hEGCs in the BAX^-/-^ mice. Notably, hilar somatostatinergic cell loss does not appear to be a factor, because NPY-ir is similar in BAX^-/-^ and wild type mice and most somatostatinergic neurons in the hilus also express NPY [101].

Remarkably, the characteristics of hEGCs in BAX^-/-^ mice were similar to the characteristics of hEGCs previously reported in animals that had SE. The characteristics of hEGCs in BAX^-/-^ mice that were similar to GCL GCs were: 1) a cell body that was small and round, 2) dendritic spines, 3) a mossy fiber axon, 4) intrinsic properties and 5) firing behavior. The characteristics that were different were 1) bipolar dendrities, and 2) spontaneous burst discharges. Taken together, the data suggest that brain insults *per se* are not the only influence on morphological and physiological development of hEGCs. Furthermore, the data suggest a relative resistance of some characteristics to perturbations like SE and *BAX* deletion.

However, there are some caveats. Not all hEGCs were studied. All intrinsic properties and physiological behavior were not assayed. Also, some characteristics that are discussed as ‘similar’ were not identical. For example, although, the fraction of hEGCs which exhibited burst discharges was similar between BAX^-/-^ mice and rodents with SE, as was the frequency of burst discharges, the complexity of some bursts was modest in BAX^-/-^ mice relative to rats after SE. For example, bursts were not as long-lasting and fewer action potentials were evoked during bursts in BAX^-/-^ mice compared to previous studies of hEGCs of epileptic rats [17,52,79]. It is an open question whether these seemingly minor differences could nevertheless have functional impact.

How would spontaneous discharges develop in hEGCs of the BAX^-/-^ mouse? In the epileptic rat, area pyramidal cells begin to develop spontaneous bursts several weeks after SE, and appear to drive hEGCs [17]. The reason for pyramidal cell bursts after SE is considered to be complex, a result of a loss of vulnerable GABAergic neurons that disinhibit pyramidal cells, sprouting of pyramidal cell axons onto other pyramidal cells so that recurrent excitatory circuitry is increased, and possibly other factors. Interestingly, spontaneous bursts of action potentials in a single pyramidal cell in a normal CA3 network can lead to bursts in area CA3 [102,103]. If recurrent excitatory circuitry was increased in the BAX^-/-^ mouse, which would seem likely due to an increased number of GCL GCs and pyramidal cells surviving into adulthood, there might be an increased likelihood that hEGCs would have a strong excitatory drive.

### II: Effect of hEGCs in the computational model of DG

#### A. Standard vs. Intermediate model: effects of adult neurogenesis

Comparison of the Standard and Intermediate models showed that the response of GCL GCs to a perforant path input pattern was relatively unaffected by the simulation of adult neurogenesis. The results are consistent with prior empirical studies suggesting that the perforant path synapses are similar anatomically, whether the synapse is on a GC that is mature or immature [1,3]. Field potential recordings in response to perforant path input are also similar, whether adult-born neurons are present or not, although differences are present if recordings are made in the GCL [104]. The model results suggest that the effects of hEGCs in the New model were not simply a result of a small (5%) population of GCs that were highly excitable, because this was a characteristic of both the Intermediate and New models.

In pattern separation, there was no detectable difference between the Standard and Intermediate models in the DG. In pattern separation in CA3 the same result occurred. However, there was a modest improvement in the Intermediate model when pattern completion was tested, particularly when the challenge was great (increased number of patterns; Figure 11C-D). These data are consistent with a modest improvement in behavioral tasks that require pattern separation when adult-born neurons are increased [105]

One reason the Intermediate model did not show a striking improvement compared to the Standard model could be related to the modeling. Prior empirical studies suggest that the primary benefit of adult neurogenesis may be either to provide continual turnover of the GC population across the lifespan, creating a “pool” of new neurons that are always available to encode new information, or to encode information about the temporal context in which new learning occurs (for review, see 106). The experiments simulated here consider a relatively short “timespan” and this may limit the ability of newborn neurons to contribute. It would be interesting in the future to examine the degree to which hEGCs, particularly in the presence of CA3 backprojections, could affect pattern storage and pattern completion along the longer timeframe considered in other computational models of adult neurogenesis [106-110].

#### B. Standard vs. New model-Summary

There were several differences between the Standard and the New model. First, there was a robust inhibitory effect of hEGCs on the firing of the GCL GC population. Second, the New model was not extremely different in its effects on GC firing when pattern number increased (Figure 9A) but there was impaired pattern separation in the DG (Figure 9B) and in CA3 (Figure 10B). Third, the New model had a nonlinear effect on pyramidal cell firing, normalizing the changes observed in the Standard and Intermediate models as the number of input patterns increased (Figure 10A). Finally, the New model performed pattern completion in CA3 much worse than the Standard model, under all conditions.

#### C. Standard vs. New model-Effects on GCL firing and pattern separation in the DG

**Figure 12:**
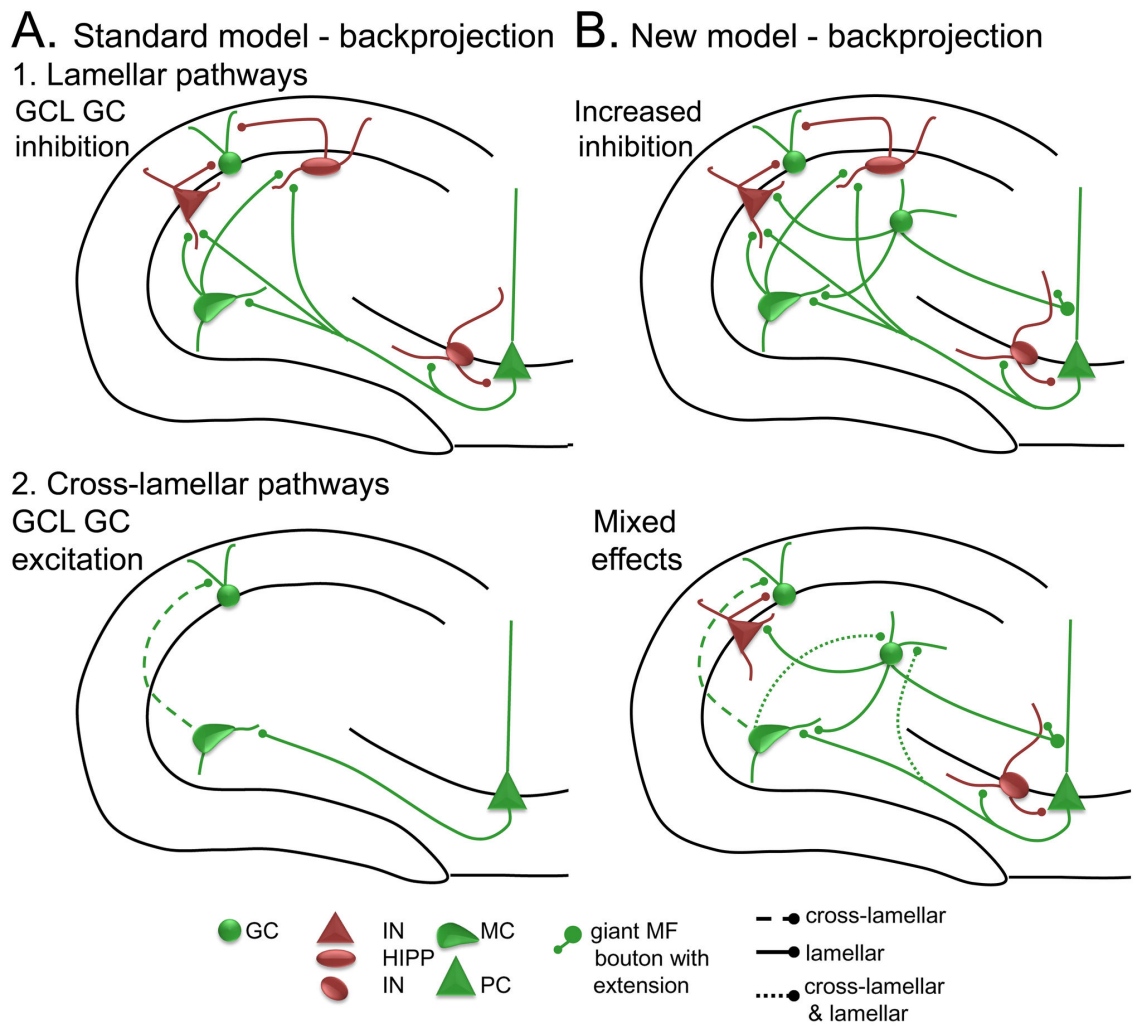
Role of the backprojection on effects of hEGCs. Schematics of the Standard (A) and New models (B), respectively, with glutamatergic (green) and GABAergic (red) components distinguished. **A**. 1. Schematics are shown that only include components of the circuitry that are driven by the backprojection. The backprojection of the Standard model inhibits GCL GCs via DG INs. 2. A schematic is used to show that the excitatory effect of the backprojection in the Standard model only occurs outside the lamella where the backprojection originated. Thus, pyramidal cells giving rise to the backprojection excite mossy cells in the same lamella, and mossy cells project outside that lamella to GCs. As in Figure 1, cross-lamellar pathways are denoted by dashed lines. **B**. 1. The backprojection of the New model is shown schematically. It gives rise to the same inhibitory projections as in A1 as well additional polysynaptic pathways mediated by hEGCs. The lamellar effects of these pathways on GCL GCs are inhibitory. Because of the additional inhibitory pathways, the New model inhibits GCL GCs more than the Standard model (as shown in Figure 8). 2. There are many cross-lamellar pathways that are activated by the backprojection in the New model, because either one segment of the pathway is cross-lamellar (axons from mossy cells to GCL GCs) or both lamellar and cross-lamellar (backprojections to hEGCs; hEGCs to mossy cells). Therefore, the backprojection has different effects in the Standard and New models. As in Figure 1, pathways that are both lamellar and cross-lamellar are designated by dotted lines.

In the New model, there was a decrease in mature (GCL) GC activity in response to an input pattern, relative to Standard and Intermediate models. In contrast, there was robust firing of hEGCs. These observations are intriguing because the model is biased against this result: because hEGCs in the model only have dendrites in the hilus, they do not receive direct input from the perforant path. The results most likely reflect the fact that, in the model, GCL GCs have strong inhibitory input which hEGCs lack, and hEGCs receive mossy fiber and pyramidal cell input that GCL GCs lack (Figure 12A–D).

Although there was a robust **percentage** of hEGCs that were active in the New model, the **total** number of active hEGCs was still fairly small (<25; Figure 8A-B). It is notable that such a small population of active hEGCs could nevertheless profoundly affect pattern separation in the DG network. The results predict that hEGCs would impair pattern separation behaviors *in vivo*. Indeed, it is known that both BAX^-/-^ mice and SE produce cognitive deficits [38,111-113]. In BAX^-/-^ mice, behavior was impaired, and the behavioral paradigm that was used is considered to depend on the entorhinal cortex, which projects to the DG [38]. The *in vivo* data are consistent with the prediction that hEGCs affect DG function *in vivo.*

The mechanism by which hEGCs impaired pattern separation in the DG network could be the small but significant decrease in GCL GC firing in the New model (Figures 8A, 9A). One might think that this result is surprising because greater quiescence of GCL GCs would seem likely to improve pattern separation; a quiescent DG is assumed to support pattern separation by allowing different subpopulations of GCs to respond to overlapping inputs. When more GCs are silent, more are available to respond to a new input. However, in the New model, the hEGCs are highly active, which means that a percentage (albeit a small percentage) of the total GC population (hEGCs + GCL GCs = total) were consistently responding to inputs and therefore not discriminating between inputs – which in turn decreases pattern separation. A similar suggestion has been made by Aimone & Gage [106] who suggest that the increased excitability of immature GCL GCs can actually reduce pattern separation.

It is also notable that activation of hEGCs decreased, instead of increased, activity of GCL GCs in the New model. The reason this is surprising is that hEGCs are glutamatergic and, in the epileptic rat, their hyperexcitability has been suggested to predispose the DG to seizure activity [14,17,25,26,27,29,49,114]. If one considers the circuitry in detail, however, both scenarios (SE and *BAX* deletion) may be correct. Simple provision of hEGCs to the network in BAX^-/-^ mice might lead to net inhibition of GCL GCs, as observed in the New model because of greater DG interneuron activation (Figure 12). However, under conditions where interneurons are reduced in number (i.e., TLE), there could be disinhibition of GCL GCs. Addition of epileptic pathology to the model, to simulate the epileptic DG, would be an interesting avenue for future work with the model.

#### D. Standard vs. New model-Critical role of the backprojection

The inhibitory effects of hEGCs on GCL GCs in the New model (Figure 8) might be due to a number of different factors, such as their placement in the hilus, which leads to direct input from the backprojection. Indeed, immature GCL GCs with similar low threshold as hEGCs (but no backprojection input) did not exert an effect on GCL GC firing or pattern separation (i.e., results from the Intermediate model). In contrast, removing the backprojection in the New model abolished the effects of hEGCs on GCL GC firing (Figure 8), suggesting this pathway was critical.

Inspection of the pathways in the models (Figure 12) suggests why removing CA3 backprojections dramatically affected the New model: in the New model, they are a relatively major source of excitatory input to hEGCs. Thus, in the New model, hEGCs do not receive perforant path input, and receive excitatory inputs only from a small number of active GCL GCs (only 1-2 per lamella in the model) and mossy cells (only 1 within the lamella in the model). By contrast, there is a relatively large number of afferents from active pyramidal cells to each hEGC (typically 10-40% of the 30 pyramidal cells in the same lamella will be active, as shown in Figure 10A).

On the other hand, the backprojection had a different effect on GCL GCs. In the Standard model, there were inhibitory effects in the same lamella and excitatory effects on GCL GCs outside the lamella (Figure 12A1-2). The inhibitory effects were mediated by interneurons in the DG. The excitatory effects were mediated by mossy cells. In the New model, there was an increase in the lamellar excitation (excitation within the same lamella) of pyramidal cells and mossy cells, driving more inhibition of local GCL GCs (Figures 8, 12B1). There was increased complexity of pathways outside the lamella (Figure 12B2), which is likely to be the reason why there was disrupted pattern separation and completion in the DG in the New model (Figure 9B).

#### E. Standard vs. New model-Effect of hEGCs on activity and pattern separation in CA3

The presence of hEGCs in the New model had a striking effect on pyramidal cells. First, pyramidal cell responses to the perforant path input were modified. In the Standard and Intermediate models, pyramidal cell firing increased as the number of stored patterns increased. However, in the New model, pyramidal cell firing was relatively constant as the number of input patterns varied. To explain this effect, we start with the Standard and Intermediate models, where pyramidal cell activity increased with the number of patterns stored, presumably because training results in strengthening of recurrent collaterals between co-active pyramidal cells. As more patterns were trained, the probability that any two pyramidal cells were co-active in at least one pattern rose, and pyramidal cell firing increased. Figure 9A shows that training as few as 100 patterns can result in activation of more than 40% of pyramidal cells. In contrast, in the New model, the activation of pyramidal cells was reduced with increased input pattern number, remaining at about 20% regardless of the number of input patterns. For low numbers of trained patterns, hEGCs probably provide additional excitatory input to pyramidal cells compared to the Standard and Intermediate models, explaining the increase in pyramidal cell firing when input pattern number is low. However, as the number of patterns rises, the same hEGCs tend to be active to all patterns, so the probability that any two pyramidal cells are both active tends to be similar, regardless of number of patterns stored.

However, this normalization comes at a “cost”: the fact that fewer distinct pyramidal cells are activated by GCL GCs in the New model reduces the effectiveness of the DG-CA3 circuit to activate different pyramidal cells in response to different patterns of perforant path input. Therefore, pattern separation fails as the number of trained patterns increases, as shown in Figure 10B. Thus, the addition of a relatively small number of hEGCs is sufficient to dramatically impair CA3 function. Again, the absence of such an effect in the Intermediate model suggests that the impaired pattern separation in the New model is specifically attributable to the abnormal location and connectivity of the hEGCs, rather than their increased excitability.

Further, pattern completion was also impaired in the New model. This could be attributed to the failure of pattern separation in the New model. If patterns are not sufficiently distinct when they are stored, then it is unlikely that “accurate” copies will be retrieved later. The net result is that the presence of a relatively small population of hEGCs, in numbers and with characteristics similar to those observed in the BAX^-/-^ mice - and in rodents that have had SE or febrile seizures - can cause a disproportionately large disruption of memory storage and retrieval in the CA3 network.

### III: Significance

HEGCs have been described in animal models and in human tissue. However, the complexity of the animal models and human disease has made it hard to understand the effects of hEGCs. The results provide a new transgenic model of hEGC formation, the BAX^-/-^ mouse which does not require severe seizures. Remarkably, hEGCs from BAX^-/-^ mice still developed characteristics that were found after brain injury. Therefore, GCs appear to develop in a very similar way if they are mislocated in the hilus, independent of the cause of the ectopic location There are certain characteristics that are plastic - dendritic development and circuit-dependent activity. In contrast, there is a relative invulnerability in intrinsic electrophysiological properties and the fundamental anatomy of the mossy fiber axon.

Although BAX^-/-^ mice were useful to study hEGCs in the absence of epilepsy, the constitutive deletion of *BAX* led to effects other than hEGC formation. Therefore, to address specific effects of hEGCs on DG-dependent behavior, computational modeling was used. The results suggest that the hEGCs have a robust effect on DG and CA3 function, even if they are a population that is only 5% of the total number of GCs in the DG. Furthermore, the effects are distinct from simply adding a population of excitable GCs, also 5% of the total number of GCs, to the GCL. The results also emphasize the importance of the backprojection to the adverse effects of hEGCs.

Given hEGCs may develop for diverse reasons, both genetic and environmental, and adverse effects may emerge even if the hEGC population is small, it seems timely to suggest hEGCs should be considered in the etiology of neurodevelopmental illnesses.

## Supporting Information

Text S1 Simulation details. The methods used for simulations are described in detail.
(DOCX)
