# Supplementary material for: The Influence of Ectopic Migration of Granule Cells into the Hilus on Dentate Gyrus-CA3 Function
Source: PLoS One. 2013 Jun 28;8(6):e68208. doi: 10.1371/journal.pone.0068208 (PMC3695928; doi:10.1371/journal.pone.0068208)
Supplement: Text S1 — The methods used for simulations are described in detail. (DOCX) [file pone.0068208.s001.docx]

**SUPPORTING INFORMATION**

**SIMULATION DETAILS**

The model includes 10 simulated lamellae. In the DG, each lamella contains 100 GC, 1 interneuron, 3 mossy cells, and 1 HIPP cell. In CA3, each lamella contains 30 pyramidal cells and 1 interneuron. Perforant path inputs are simulated as a vector of 200 axons, each of which can be 1 ("active" or "firing") or 0 ("inactive" or "not firing"). A "trial" begins when a new subset of perforant path inputs are activated, and this pattern (an "input pattern") is presented to the DG and CA3, and ends with a readout of the responses of GCs and pyramidal cells to that input pattern. The responses of GCs and pyramidal cells are designated as either suprathreshold (an action potential, output=1) or subthreshold (no action potential; output=0). There are two output patterns, one representing DG output, which is a vector of 0’s and 1’s representing the action potentials produced by the GCs, and one representing CA3 output, which is a similar vector representing the action potentials produced by the pyramidal cells.

**DG Network**

At the start of each simulation run, a new set of DG cells is constructed; in the Standard model, all GCs are placed in the GCL and are designated as "mature GCs." In the Intermediate model, all GC are placed in the GCL but a fraction (5%) are randomly designated as "immature GCs." In the New model, a fraction (5%) of GCs are randomly designated as "hilar EGCs" (hEGCs) and placed in the hilus. Immature GCs and hEGCs differ from mature GCL GCs in their connectivity patterns (see below) and firing threshold, defined as Θ. Specifically, Θ[*g*]=0.75 for mature GCs *g* and Θ[*g*]=0.70 for immature (including hilar) GCs *g*. For all (mature, immature and hilar) GCs *g*, resting potential Vrest[*g*]= -0.3.

Next, a connectivity matrix is formed according to the following rules; each presynaptic cell *x* connects with some probability to each postsynaptic cell *y*, and if a connection exists, the synaptic weight *w[x,y]* is initialized from the normal distribution *U*[0..1).

1. Each perforant path input *e* projects with probability 0.2 to each GCL GC *g* (across all lamellae) with *w[e,g]*. Note that hEGCs do not receive direct perforant path input.
2. In addition, for each hEGC *g,* each GCL GC *g2* (in all lamellae) projects to *g* with probability 0.2, with weight *w[g2,g]*; each pyramidal cell *c* (in all lamellae) projects to hEGC *g* with probability 0.2, with weight *w[c,g]*.
3. Each perforant path input *e* also projects with probability 0.2 to each HIPP cell *h* (across all lamellae), with weight *w[e,h]*. Each HIPP cell *h* in lamella *l* also receives input from all MCs *m* in *l*, with weight *w[m,h]*, and from all pyramidal cells in *l*, with weight *w[c,h]*.
4. Each HIPP cell *h* in lamella *l* projects to all GCL GCs (not hEGCs) *g* in *l*, with weight *w[h,g]*.
5. Each mossy cell *m* in lamella *l* receives input from all GCs (GCL GCs and hEGCs) *g* in *l*, with weight *w[g,m]*, and from all pyramidal cells in *l*, with weight *w[c,m]*.
6. Each mossy cell *m* in lamella *l* projects with probability 0.2 to each GCL GC *g* in other lamellae (not in *l*) with weight *w[m,g]*, and with probability 0.2 to each hEGC *g* across all lamellae (including *l*), with weight *w[m,g]*.
7. The DG IN *i* in lamella *l* receives inputs from all GCs *g* in *l*, with weight *w[g,i]*, from all mossy cells *m* in *l*, with weight *w[m,i]*, and from all pyramidal cells in *l*, with weight *w[c,i]*.
8. The DG IN *i* in lamella *l* projects to all GCL GCs (not hEGCs) in *l*, with weight *w[g,i]*.

When an input pattern generated by the perforant path is received by the DG, cells compute activity in the following sequence:

1. First, each GC *g* responds to perforant path inputs and, in the case of hEGCs, to mossy fiber, and CA3 backprojection inputs:

$$activ[g]=Vrest[g]+\sum_{e} activ[e]*w[e,g]+\sum_{g2\neq g} activ[g2]*w[g2,g]+\sum_{c} output[c]*w[c,g]$$

for all perforant path inputs *e*, other GCs *g2*, and pyramidal cells *c*.

2. Next, each DG IN *i* calculates activity based on the maximally-active GCL GC *g* in *l* and backprojections from pyramidal cells *c*:

$$activ[i]=\max_{g } (activ[g])+K_{bp}\sum_{c} output[c]*w[c,i]$$

where *K_bp_*=0.01. Note that hEGCs do not project to DG INs.

3. Each DG IN *i* then feeds back to inhibit GCL GCs *g* in the same lamella, so that all but the most-active GCs are silenced:

$$activ[g]-=K_{DG\_IN}*activ[i]$$

where *K_DG_IN_*=0.9. Note that DG INs do not project to hEGCs.

4. Next, each mossy cell *m* in lamella *l* receives inputs from GCs (GCL GCs and hEGCs) *g* in *l* and from pyramidal cells in *l*:

$$activ[m]=\sum_{g} activ[g]*w[g,m]+K_{bp}\sum_{c} output[c]*w[c,m]$$

where *K_bp_=0.01*.

5. Each mossy cell *m* in lamella *l* then projects to a subset of GCL GCs *g* (outside *l*) and hEGCs *g* (across all lamellae), as specified in the connectivity matrix:

$$activ[g]+=\sum_{m} activ[m]*w[m,g]$$

6. Next, each HIPP cell *h* in lamella *l* receives perforant path inputs *e*, mossy cells *m* within *l*, and pyramidal cells *c* in *l*:

$$activ[h]=\sum_{e} activ[e]*w[e,h]+\sum_{m} activ[m]*w[m,h]+K_{bp}\sum_{c} output[c]*w[c,h]$$

where *K_bp_*=0.01.

7. Each HIPP cell *h* in lamella *l* then projects to all GCL GCs *g* in *l*, as specified by the connectivity matrix:

$$activ[g]-=K_{HIPP}\sum_{h} activ[h]*w[h,g]$$

where *K_HIPP_*=0.1. Note that HIPP cells do not project to hEGCs.

8. Finally, for all GCs (both GCL GCs and hEGCs) *g*, *g* fires (produces output=1) if net activation, passed through a sigmoidal function, exceeds the firing threshold:

*If f(active[g])> θ[g] then output [g]=1 else output [g]=0*

where$f(x)=1/(1+e^{-(2x-1)})$.

The vector of output values is the external output of the DG network (the mossy fibers) to the current pattern of perforant path inputs; these project to the CA3 network and are also used to assess pattern separation, etc., in the DG network.

**CA3 Network**

At the start of each simulation run, a set of CA3 cells is constructed with a new connectivity matrix (see below). For all pyramidal cells, Vrest[*c*]= -0.3 and Θ[*c*]=0.5. There are no differences in the CA3 network between the Standard, Intermediate, and New models, except for their connectivity with the DG network (as described above). The CA3 network can be run in either PATTERN STORAGE mode, when mossy fiber afferents from the DG are enabled and function as forcing or teaching inputs, or PATTERN COMPLETION mode, when mossy fibers are silenced but recurrent collaterals among pyramidal cells are enabled.

Each connectivity matrix is formed according to the following rules:

1. Each perforant path input *e* projects to 2% of pyramidal cells *c* (across all lamellae) with weight *w[e,c]*=[0..1) and to the remaining *c* with *w[e,c]*=0.
2. Each pyramidal cell *c* projects to 4% of other pyramidal cells *c2≠c* (across all lamellae) with weight *w[c,c2]*=[0..1) and to all remaining pyramidal cells *c2≠c* with weight *w[c,c2]*=0.
3. Each pyramidal cell *c* in lamella *l* projects to the CA3 IN *i* in *l* with weight *w[c,i]*=1 and *i* projects back to *c* with *w[i,c]*=1.
4. Each GC (GCL GC and hEGC) *g* in lamella *l* projects to a single random pyramidal cell *c* in *l*, with weight *w[g,c]*=1 and to the remaining pyramidal cells with weight *w[g,c]*=0; each GC *g* in *l* also projects to the CA3 IN *i* in *l*, with weight *w[g,i]*=0.1.

When a perforant path input pattern is presented to CA3, cells compute activity in the following sequence:

1. First, each pyramidal cell *c* responds to perforant path inputs *e*:

$$activ[c]=Vrest[c]+\sum_{e} activ[e]*w[e,c]$$

1. If the network is in PATTERN COMPLETION mode, then recurrent collaterals from other pyramidal cells *c2* also provide input:

$$activ[c]+=\sum_{c2\neq c} output[c2]*w[c2,c]$$

1. Otherwise, if the network is in PATTERN STORAGE mode, then mossy fibers from GCs *g* also provide input to *c*:

$$activ[c]+=K_{MF\to PYR}\sum_{g} output[g]*w[g,c]$$

where *K_MF→PYR_*=100 (i.e. activity of one mossy fiber input to *c* is generally sufficient to cause *c* to fire).

1. Next, the CA3 IN *i* in each lamella l calculates activity based on the maximally-active pyramidal cell *c* in *l*:

*active [i] =* max *(active[c])*

*c*

1. If the network is in PATTERN STORAGE mode, then the mossy fibers from GCs *g* also provide input to *i*:

$$activ[i]+=K_{MF\to IN}\sum_{g} output[g]*w[g,i]$$

where *K_MF→IN_*=0.1.

1. The IN *i* in *l* then feeds back to inhibit pyramidal cells *c* in the same lamella, so pyramidal cells that are less active are silenced:

*active[c]- = 0.5*active[i]*

1. Finally, each pyramidal *c* fires (produces output=1) if net activation, passed through a sigmoidal function, exceeds the firing threshold:

*If f(active [c]) >θ [c] then output [c] = 1 else output[c]=0*

where *f(x)* is defined as for the DG network.

The vector of pyramidal cell outputs forms the backprojection to the DG network and is also used to assess pattern completion, etc., in the CA3 network.

**DG-CA3 Interaction**

For each simulation run, DG and CA3 are initialized as described above. Next, a specified number (*N_Pat_*) of input patterns are generated, each with a specified percentage (or density, *d*) of elements that are active (1) with the remainder inactive (0). In the simulations reported here, the DG-CA3 model is given 5 training passes through the set of *N_Pat_* input patterns, followed by a pattern completion test.

During each training pass, for each pattern *p*, the corresponding values are presented as perforant path inputs to the DG and CA3 networks. First, CA3 (operating in PATTERN COMPLETION mode) responds to the input; this is repeated 5 times, allowing information (i.e., action potentials) to propagate along the recurrent collaterals, and the CA3 network to settle into a stable pattern of output. At this point, the output from CA3 is provided as backprojections to the DG network, and the DG network responds to this and the perforant path input, producing output from the GCs. Finally, CA3 responds to the input once more in PATTERN STORAGE mode, in which the mossy fiber projections from DG are active but CA3 recurrent collaterals are suppressed. Synaptic weights in CA3 are then modified, using a rule that approximates observed mechanisms of LTP/LTD (per [[115](#_ENREF_115)]), with learning rate *LR*=0.5:

1. Weights from perforant path input *e* to pyramidal cells *c* in lamella *l* are updated: *w[e,c]+=LR*output[c]*(activ[e]-w[e,c])*

2. Weights of recurrent collaterals from pyramidal cell *c2* to pyramidal cell *c* are updated: *w[c2,c]+=LR*output[c]*(output[c2]-w[c2,c])*

Once all training passes are completed, a pattern completion test may be performed. Here, for each trained pattern *p*, the CA3 network runs (in PATTERN COMPLETION mode) and the output from the pyramidal cells to *p* are noted as a vector *R[p*]. Note that *R[p]* consists of 300 binary values, each specifying whether one of the 300 pyramidal cells produces output 0 or 1 in response to *p*. Next, for each trained pattern *p*, 10 "distorted" versions *p'* are generated that vary from *p* by setting a fixed percentage (0%, 10% ... 90%) of the active elements in *p* to 0. Each distorted pattern *p'* is then presented to CA3 which produces output *R[p']*. The overlap between *R[p]* and *R[p']* is defined as the number of bits with the same value (i.e., the number of pyramidal cells that produce the same output to both *p* and *p'*). If the overlap between *R[p']* and *R[p]* is greater than the overlap between *R[p']* and the response *R[q]* to any other stored pattern *q*, then the CA3 network has correctly performed pattern completion.

For Figure 8, the results reflect one representative simulation run for each model (Standard, Intermediate or New). In Figures 9-11, each value in the graphs represents the mean of 10 simulation runs. Simulation results vary from run to run because there are differences in initial conditions - specifically the connectivity matrix and the exact sequence of the trained patterns. Thus, results are presented as a mean with a standard error of the mean, which represents the expected degree to which results vary across simulation runs.
